# GGCX-Associated Phenotypes: An Overview in Search of Genotype-Phenotype Correlations

**DOI:** 10.3390/ijms18020240

**Published:** 2017-01-25

**Authors:** Eva Y. G. De Vilder, Jens Debacker, Olivier M. Vanakker

**Affiliations:** Center for Medical Genetics Ghent, Ghent University Hospital, Ghent 9000, Belgium; eva.devilder@ugent.be (E.Y.G.D.V.); jensm.debacker@ugent.be (J.D.)

**Keywords:** gamma-carboxylation, GGCX, cutis laxa, pseudoxanthoma elasticum, VKCFD1, elastic fibers

## Abstract

Gamma-carboxylation, performed by gamma-glutamyl carboxylase (GGCX), is an enzymatic process essential for activating vitamin K-dependent proteins (VKDP) with important functions in various biological processes. Mutations in the encoding *GGCX* gene are associated with multiple phenotypes, amongst which vitamin K-dependent coagulation factor deficiency (VKCFD1) is best known. Other patients have skin, eye, heart or bone manifestations. As genotype–phenotype correlations were never described, literature was systematically reviewed in search of patients with at least one *GGCX* mutation with a phenotypic description, resulting in a case series of 47 patients. Though this number was too low for statistically valid correlations—a frequent problem in orphan diseases—we demonstrate the crucial role of the horizontally transferred transmembrane domain in developing cardiac and bone manifestations. Moreover, natural history suggests ageing as the principal determinant to develop skin and eye symptoms. VKCFD1 symptoms seemed more severe in patients with both mutations in the same protein domain, though this could not be linked to a more perturbed coagulation factor function. Finally, distinct GGCX functional domains might be dedicated to carboxylation of very specific VKDP. In conclusion, this systematic review suggests that there indeed may be genotype–phenotype correlations for GGCX-related phenotypes, which can guide patient counseling and management.

## 1. Introduction

**Figure 1:**
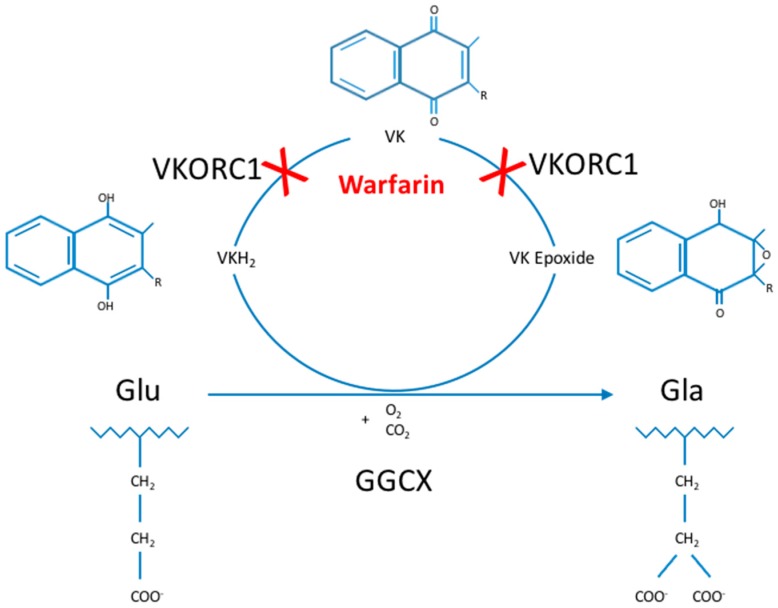
The Vitamin K cycle. Glu-residues are gamma-carboxylated by GGCX to Gla-residues, an enzymatic process using VKH_2_, O_2_ and CO_2_ as cofactors. During this process, VKH_2_ is oxidized to VK epoxide, which is then reduced to VK and in a second reduction step to VKH_2_ by VKORC1. Then, VKH_2_ can be reused, the reason for which this process is called the VK cycle. Gamma-carboxylation is only performed in VKDP and is essential for their activation and downstream functioning in multiple biological processes, such as blood clotting, bone formation, inflammation and cell proliferation. Warfarin inhibits the VK cycle by preventing VK reduction. C: carbon; GGCX: gamma-glutamyl carboxylase; Gla: gamma-carboxyglutamate; Glu: glutamate; H: hydrogen; O: oxygen; R: attached hydrogen or a hydrocarbon side chain of any length; VK: vitamin K (quinone); VKDP: VK-dependent proteins; VKH_2_: vitamin K hydroquinone; VKORC1: vitamin K epoxide reductase complex, subunit 1.

The gamma-glutamyl carboxylase enzyme (GGCX) catalyzes the conversion of specific glutamate (Glu) residues to gamma-carboxyglutamate (Gla) residues, a process called gamma-carboxylation [1]. This posttranslational modification process uses vitamin K (VK) as an essential cofactor and is part of the so-called VK cycle (Figure 1) [2]. Gamma-carboxylation is essential in the activation and proper functioning of multiple VK-dependent proteins (VKDP), the most well-known of which are involved in blood clotting, including coagulation factors (FII, FVII, FIX and FX) and natural anti-clotting agents (protein C, protein S (ProS; OMIM*176880) and protein Z). Moreover, GGCX catalyzes gamma-carboxylation of other VKDP, involved in various biological processes such as inflammation (e.g., ProS, and gla-rich protein (GRP)), bone formation (osteocalcin (OC; OMIM*112260)), cell proliferation (growth arrest-specific 6 (Gas6; OMIM*600441)) and soft tissue mineralization (matrix gla protein (MGP; OMIM*154870)) [3,4]. Finally, several VKDP have a currently unknown function (proline-rich gla proteins (PRGP), and transmembrane gla proteins (TMG)) [1,2,5,6,7].

The GGCX enzyme is encoded by the *GGCX* gene, located on the reverse strand of chromosome 2p11.2 (chromosomal position in assembly GRCh38.p7: 85,544,723–85,561,509). The gene is not considered to be polymorphic, as, according to the gnomAD database (combining data from the ExAc and 1000 genomes databases), of the 409 exonic variants (missense and loss-of-function) that have been identified in the *GGCX* gene, only two variants, i.e., rs699664 and rs6173310, have an allele frequency of >0.0001 (Table S1) [8]. 

**Figure 2:**
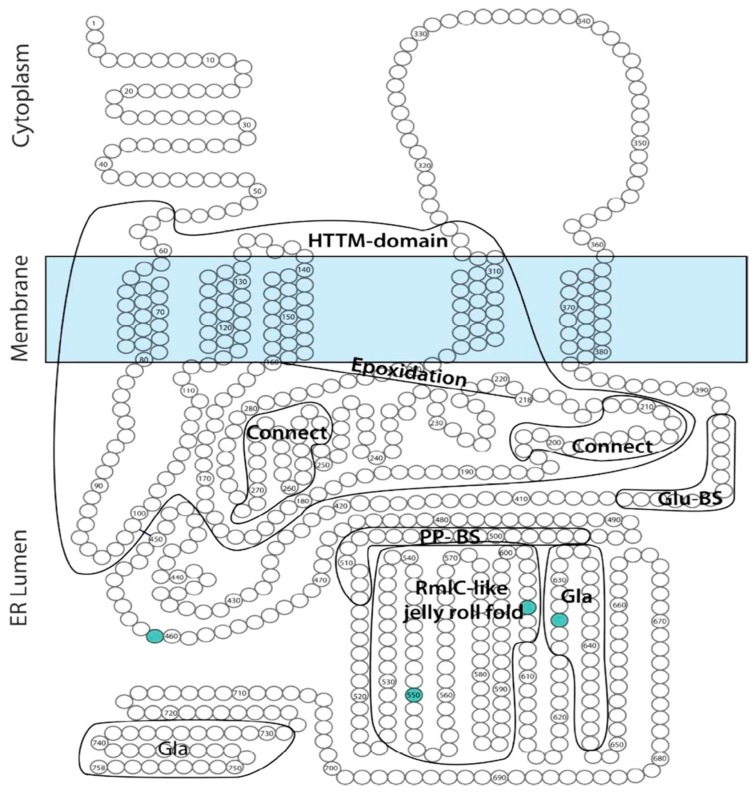
Predicted gamma-glutamyl carboxylase (GGCX) topology. Adapted from [9]. This figure gives an overview of the predicted localization of known or predicted GGCX domains on a GGCX topology model. Green circles depict amino acid residues which undergo glycosylation. Connect: hydrophobic domains important for interaction with vitamin K. ER: endoplasmic reticulum; HTTM: horizontally transferred transmembrane domain; Gla: gamma-carboxyglutamate; Glu-BS: glutamate binding site; PP-BS: propeptide binding site; RmlC: deoxythymidine-6-deoxy-d-xylo-4-hexulose 3,5 epimerase (EC5.1.3.13).

*GGCX* has 10 transcripts, of which the longest is NM000821.6 (ENST00000233838.8; Uniprot P38435), comprising 15 exons and 7569 nucleotides. The protein encoded by this transcript is a 94 kDa, 758 amino acid (AA) transmembrane protein, expressed ubiquitously throughout the body and localized on the lipid membrane of the endoplasmic reticulum (ER). The N-terminal part of GGCX is localized in the cytoplasm, followed by 5 transmembrane domains (TMD), and the C-terminal portion is localized in the ER lumen (Figure 2). To date, the crystal structure of the GGCX enzyme is still incompletely resolved [9].

GGCX has multiple highly conserved domains (Figure 2), including the horizontally transferred transmembrane domain (HTTM—AA 56–315), spanning the first four TMDs, the function of which is currently unclear in humans [10]. Interestingly, in multiple species such as eukaryotes, bacteria and archae, the HTTM-domain seems to play an important role in VK-dependent carboxylation [11]. Within the TMD, the proline-residue at position 378 in TMD5 is proposed to play an important role in the correct orientation of GGCX; replacing that proline by a leucine leads to an important decrease in the formation of a disulfide bond in the protein, which is an important posttranslational modification step (see below) [12]. However, apart from the disulfide bond other factors must play a role in GGCX orientation, as even with a removal of the disulfide bond and a complete cleavage of GGCX between TMD4 and TMD5, the GGCX protein domains remain close together. Tie et al. suggested that an interaction between TMD2 and TMD5 could play an important role in this process [12]. Other important functional domains in the GGCX enzyme are the propeptide binding site (most recently proposed to be localized at AA 491–507), suggested as the primary location of interaction between GGCX and its substrates, and the glutamate binding site (AA 393–404), which interacts with the Glu-containing regions of VKDP, a necessary step for gamma-carboxylation. Interestingly, L394 and W399, which are localized in this predicted glutamate binding region, seem to play a role in polypeptide binding by GGCX, hereby stimulating the connection between the propeptide binding sites and the glutamate binding sites, thus facilitating gamma-carboxylation [9,13,14,15]. GGCX is further predicted to contain an RmlC (deoxythymidine-6-deoxy-d-xylo-4-hexulose 3,5 epimerase; EC5.1.3.13)-like jelly roll fold, comprising a double-stranded beta-helix jelly roll fold as is identified in RmlC, from AA 526 until 607 [11]. The function of this domain is however currently unclear. 

Finally, GGCX also undergoes posttranslational modifications, such as glycosylation of 4 asparagine residues (AA 459, 550, 605 and 627) and the formation of a disulfide bond (between cysteine-residue 99 and 450), which stabilizes the protein leading to a more efficient enzymatic function [16,17,18]. GGCX further has 2 autocarboxylating Gla-domains, suggested to be localized at AA 625–647 and 729–758 in the C-terminal region of the enzyme in the ER lumen. Possibly, these Gla-domains have a yet undiscovered role in other processes than VKDP gamma-carboxylation [19].

In 1998, *GGCX* mutations were first linked to human disease by Brenner et al. in four patients with a combined deficiency of all VK-dependent blood coagulation factors (factor II, VII, IX, X and ProS and protein C) due to a homozygous missense mutation in the *GGCX* gene [13]. The disease was coined VK-dependent clotting factor deficiency-1 (VKCFD1, OMIM#277450), an autosomal recessive disorder, characterized by a mild to severe bleeding tendency and a moderate predisposition to thrombotic events [13,20]. VKCFD1 was shown to be associated with skeletal (midfacial hypoplasia, reduced bone mass, chondrodysplasia punctata) or cardiac abnormalities (patent ductus arteriosus Botalli, septal closure defects) in some patients [13,21,22,23,24,25,26,27,28,29,30]. Next to VKCFD1, a second autosomal recessive coagulation factor deficiency exists, VKCFD2 (OMIM#607473), caused by *VKORC1* (vitamin K epoxide reductase complex, subunit 1; OMIM*608547) mutations and is also characterized by a deficiency of all VK-dependent clotting factors. In contrast to VKCFD1, this general deficiency can usually be reversed completely using low doses of VK (ca. 5–10 mg/week) [31,32]. Skeletal abnormalities (in particular osteoporosis) have been described in VKCFD2 patients [33], but no cardiac involvement has been identified yet. 

**Figure 3:**
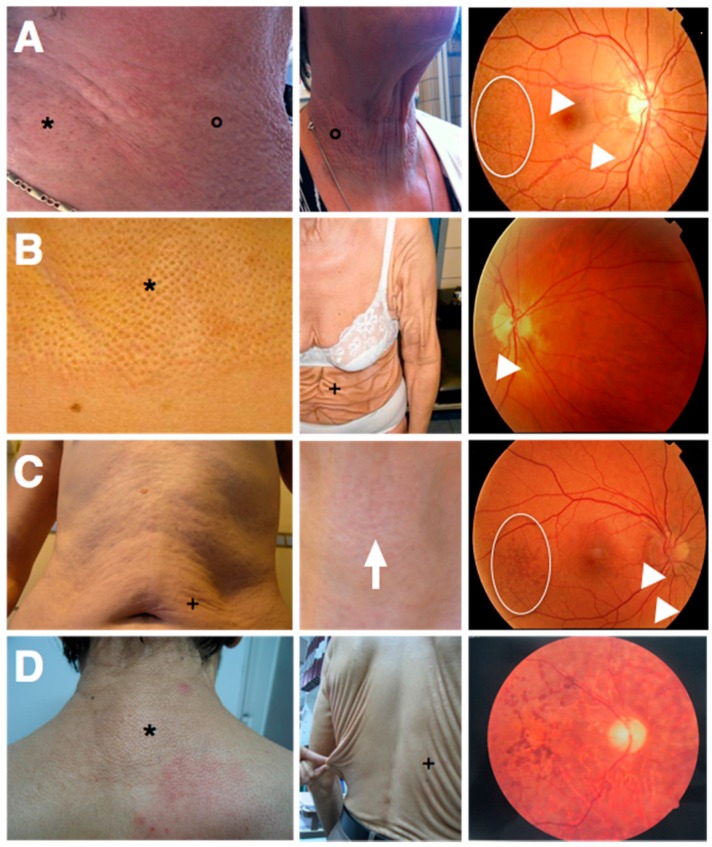
Skin and eye symptoms of the known gamma-glutamyl carboxylase (GGCX)-related disease entities. In each panel, two photos depict different aspects of the respective skin phenotypes (left and middle), the right image shows a fundus typical for the disease entity: (**A**) PXE; (**B**) PXE-like disorder with combined coagulation factor deficiency; (**C**) patient with PXE/PXE-like overlap; and (**D**) PXE-like syndrome with pigmentary retinopathy. *: yellowish skin papules, °: skin plaques; arrowheads: angioid streaks; spherical diagram: peau d’orange; +: skin loosening and excessive skin folds; arrow: reticular rash; PXE: pseudoxanthoma elasticum; PXE-like: PXE-like disorder with combined coagulation factor deficiency.

More recently, biallelic *GGCX* mutations were shown to cause a phenotype characterized by not only VKCFD1 but also elastic fiber (EF) mineralization and fragmentation, leading to loss of skin elasticity and loosening of the skin with a cutis laxa appearance. In the original seven patients, the phenotype was demonstrated to be similar to but more severe than the skin features in pseudoxanthoma elasticum (PXE; OMIM#264800), an autosomal recessive ectopic mineralization disorder. The disease was therefore called PXE-like disorder with combined coagulation factor deficiency (OMIM#610842). Classic PXE is caused by EF mineralization in soft tissues due to biallelic *ABCC6* (ATP-binding cassette, subfamily C, member 6; OMIM*603234) mutations and features yellowish skin papules and plaques in flexural areas (although in more severe cases an increase in skin laxity may occur), ophthalmological symptoms (asymptomatic peau d’orange and angioid streaks and in more advanced stages subretinal neovascularization, bleeding and scarring leading to legal blindness when untreated) and cardiovascular symptoms (peripheral artery disease, cardiac diastolic dysfunction) (Figure 3A) [34,35]. Strikingly, at first the seven patients with the PXE-like syndrome had typical dermal EF calcifications leading to yellowish papules but later progressively developed excessive skin folds, not only confined to flexural areas (Figure 3B) [36]. In contrast to PXE, cardiovascular symptoms were absent and the retinopathy much milder, with mainly asymptomatic lesions (peau d’orange and angioid streaks) [36]. Since the original report, additional patients have been identified with a similar phenotype [21,37]. 

In 2011, we described a patient with a phenotype intermediate to PXE and the PXE-like syndrome (Figure 3C). This boy had developed excessive skin folds, typical for the PXE-like syndrome, around the age of 10, which at first were confined to the abdomen, but later progressively affected the axillae, upper arms and elbows. Prior to the development of the skin folds, the skin had appeared to have an inflamed, reddish aspect, but the skin aspect was not suggestive for an acquired post-inflammatory form of cutis laxa [38]. Upon clinical inspection, a very mild yellowish reticular rash, typical for PXE, was identified in the frontal neck. Further clinical workup included fundoscopic imaging, which revealed peau d’orange and angioid streaks, abdominal ultrasound showing renal microcalcifications, and normal coagulation tests (normal activated partial thromboplastin time (aPTT) and prothrombin time (PT)). Moreover, histological, biochemical and immunohistochemical characteristics were intermediate to PXE and the PXE-like syndrome. This patient was identified with compound heterozygous *ABCC6* mutations as well as a functional single nucleotide polymorphism (SNP) in the *GGCX* gene [39].

In 2014, Kariminejad et al. described 14 patients from two unrelated families with a PXE-like skin phenotype (cutis laxa) and a pigmentary retinopathy, caused by biallelic mutations in the *GGCX* gene (Figure 3D). These patients all developed progressive vision loss with night blindness in early childhood and yellowish skin papules on the back, the lateral sides of the neck, the chest and the flexural body areas between the ages of 11 and 25 as well as an unusually loose skin on the trunk, which gradually worsened and in later stages also affected the upper limbs. Ophthalmological workup, including an electroretinogram, identified non-detectable rod responses or rod responses with reduced amplitude and prolonged implicit time, compatible with a pigmentary retinopathy, but failed to show any PXE-related eye symptoms (such as angioid streaks, peau d’orange or subretinal hemorrhage). A similar phenotype was seen in all affected patients, varying only in time of onset of the eye and skin symptoms. An echocardiography showed no abnormalities. Interestingly, none of these patients had VKCFD1 [40].

Next to the presence of phenotypes caused by mutations in the *GGCX* gene, the modifying effect of *GGCX* variants on the response to warfarin treatment (amongst others on dose and time in therapeutic range) has been suggested repeatedly in the past and has been extensively studied. However, the results of these association studies are ambiguous, seem to be population-dependent and cannot be seen separately from variants in other genes, relevant for warfarin metabolism. Hence, the effects of these *GGCX* variants fall beyond the scope of this systematic review [41,42,43,44]. 

The variability of the GGCX-related phenotypes, as illustrated above and in Table 1, is striking while their correlation with the underlying genotypes has to date remained unclear. Therefore, we explored the possibility of genotype–phenotype correlations between *GGCX* mutations and the different GGCX-related diseases.

## 2. Results

### 2.1. Article Selection

**Figure 4:**
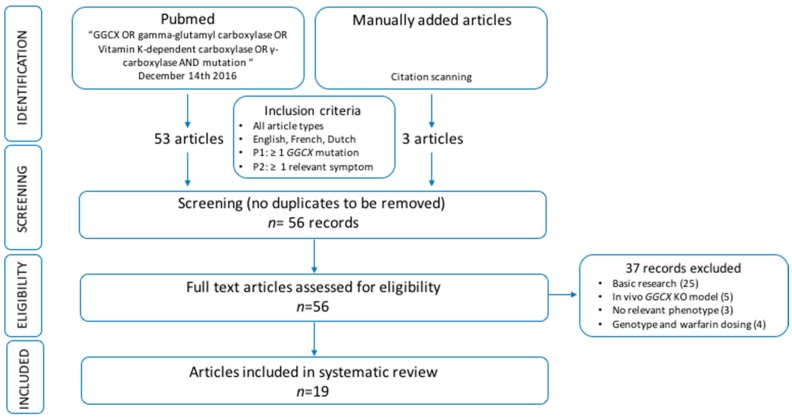
Overview of the pipeline used for the systematic search of the literature. GGCX: gamma-glutamyl carboxylase, KO: knockout, *n*: number, P1: first patient criterium, P2: second patient criterium.

Fifty-three articles were identified through a systematic Pubmed search of which 37 did not meet the inclusion criteria: 25 papers described basic research (GGCX, gamma-carboxylation and VKDP mechanisms of action; GGCX protein structure and function determination); five articles described in vivo *GGCX* knockout models; three papers mentioned no relevant phenotypes; and four assessed genotype-induced variability on warfarin dosing. Further, three papers, all meeting the inclusion criteria, were manually added through reference scanning of the included papers from the Pubmed search. In total 19 papers were included for this systematic review. The selection process followed the Preferred Reporting Items for Systematic Reviews and Meta-Analysis Protocols (PRISMA-P) and is shown in Figure 4 [45]. An overview of all reviewed publications can be found in Table S2.

### 2.2. Patient Data Extraction

Fifty individual patients with at least one *GGCX* mutation and minimally one relevant GGCX-related phenotype were withheld from the included papers. Among these, three patients with one *GGCX* mutation and mono- or biallelic *ABCC6* mutations were excluded from the analysis because in these patients the dermatological phenotype cannot be analyzed unambiguously, as both *ABCC6* and *GGCX* mutations may lead to skin symptoms belonging to the same spectrum [37,46]. Thus, 47 patients were withheld for the analysis (P1–P47; Table 2), encompassing 28 individual probands (P1, P5, P7, P8, P10, P12–P23, P25–P30, P36, P41, P44, P46, P47). Nine patients were described twice in literature (patients 10 and 11 by Goldsmith et al. and Li et al.; patient 13 by Rost et al. in 2004 and 2006; patients 16–20 by Vanakker et al. in 2007 and Watzka et al. in 2014; patient 21 by Rost et al. and Watzka et al.) [21,23,36,47,48,49]. Patient characteristics (age, sex, ethnicity and age of first symptoms) of the 47 patients included in the analysis can be found in Table 2.

### 2.3. Genotype Analysis

Mutations were annotated according to transcript NM000821.6 in 12 out of 19 papers. Five publications used reference sequence BC013979, in which +1 is located at the −28 position of NM000821.6 [22,36,37,48,50]. Therefore, the annotation of these mutations was corrected accordingly (Table 3). The genotypes of P18 and P19 were already corrected by Watzka et al., but for one mutation, i.e., c.1339G>T, the mutation remained incorrectly annotated and was corrected in this paper [21,36]. Two publications used unspecified reference sequences, leading to different cDNA-annotations, but the same protein annotations; cDNA annotations were updated to reference sequence NM0000821.6.

Thirty-two different mutations were identified, 14 of which were located in the HTTM-domain (amongst others one in TMD1, two in TMD1 and -2, one in TMD2, two in TMD3 and one in TMD4), one mutation affected the N-terminal region and the first part of the HTTM-domain (TMD1), one mutation was located in TMD5, three at or near the glutamate binding site, six at or near the propeptide binding site, three mutations were found in the RmlC-like jelly roll fold, two mutations near the propeptide binding site and RmlC-like jelly roll fold and one near the most C-terminal autocarboxylated Gla-domain. One mutation was a 14 bp deletion in intron 1, not leading to a change at the protein level (Table 2). This mutation was proposed to influence expression of GGCX in the affected patients, as the deletion destroyed a reverse palindromic sequence (TTGAGGCAA), often associated with *cis*-acting elements (involved in protein expression) [27]. Interestingly, in P47, harboring a homozygous splice site mutation leading to the deletion of exon 2, paternal uniparenteral disomy was identified [30].

### 2.4. Exploring Possible Genotype-Phenotype Correlations in GGCX-Related Phenotypes

*GGCX* mutations were identified mainly in combination with five distinct phenotypes: ophthalmological, dermal, cardiac and osseous symptoms, and coagulation abnormalities. These phenotypes can be related to PXE (ophthalmological and dermatological symptoms), VKCFD1 or the fetal warfarin or DiSaia syndrome (cardiac, skeletal and facial abnormalities). 

#### 2.4.1. Cardiac Phenotype

Eight patients were reported to have congenital heart defects, i.e., a persistent ductus arteriosus Botalli (P13, and P27), septal closure defects (P5, P6, P25, P28, and P47), Wolff–Parkinson–White syndrome (P47) and a congenital supravalvular pulmonary stenosis and peripheral pulmonary artery stenosis (P8) (Table 4). Apart from P8 and P26, all patients also had facial dysmorphisms and/or skeletal features. P8, the only patient with confirmed congenital pulmonary stenosis, has a sister (P9) carrying the same *GGCX* genotype with no cardiac phenotype.

Seven different genotypes and in total 11 different *GGCX* mutations were identified in the eight patients with a cardiac phenotype; and 7/11 *GGCX* mutations were located in the HTTM domain (1 in TMD1 and -2, one in TMD3, and one in TMD4), one mutation affected the N-terminal region and the first part of the HTTM-domain (TMD1), one mutation was localized in the RmlC-like jelly roll fold, one near the propeptide binding site and one near the C-terminal Gla domain. The mutation p.(W157R) was identified in three patients, of whom two siblings with p.(T591K) on the other allele and one unrelated patient with c.2085-5T>C as the second mutation. The genotype p.(V255M); p.(S300F) was identified in one patient (P8) with a cardiac problem and in this patients’ sibling (P9) with no cardiac abnormalities. Overall, all patients with a cardiac defect had at least one mutation in the HTTM domain. 

#### 2.4.2. Dermatological Phenotype

Twenty-three patients had dermatological PXE-like symptoms, i.e., yellowish papules and excessive skin folds reminiscent of cutis laxa (Table 5). Affected regions included the neck and flexural areas (amongst others the axillae), but also the trunk, groins and/or chest. The age of onset of the skin lesions was under the age of 10 in one patient, between 10 and 20 years in 17 patients, above the age of 20 in two patients and not mentioned in three adult patients with skin symptoms. Overall, in this cohort of 47 patients, 21/24 patients older than 18 years had PXE-like skin symptoms. P17 already had skin symptoms at three years of age, which became more severe during puberty. 

Nine different genotypes were associated with skin symptoms, encompassing 11 different *GGCX* mutations. Five mutations were located in the HTTM domain, four near the propeptide binding site, one in the glutamate binding site, one in the RmlC-like jelly roll fold and one in TMD5. In P17 and P18, only one *GGCX* mutation could be identified. In all other patients, compound heterozygous or homozygous *GGCX* mutations were present.

#### 2.4.3. Ophthalmological Phenotype

Eighteen patients were diagnosed with ophthalmological symptoms, including isolated angioid streaks (P10, P16, and P17), angioid streaks and peau d’orange (P11, and P18) and a pigmentary retinopathy of variable severity (P31–P43) (Table 6). All 18 patients with ophthalmological symptoms also had PXE-like skin symptoms. 

The eye symptoms were associated with six different genotypes comprising eight different mutations: four mutations were located in the HTTM domain, three near the propeptide binding site and one in TMD5. In three of the six genotypes associated with eye problems in this cohort, at least one mutation was located N-terminally of the propeptide binding site (i.e., p.(R476H), p.(R476C), p.(W493S)). The arginine-residue at position 476 is part of a highly conserved seven AA-long sequence (N-ND**R**FQQR-C) and the tryptophan-residue at position 493 is highly conserved among different species (11/11) (Table S3). The *GGCX* genotype p.(F73_G125del); p.(F73_G125del), leading to a homozygous deletion of exon 3, is the only genotype that is associated with a pigmentary retinopathy in all affected patients (P31–P43). Interestingly, all examined patients in these families were affected by this retinopathy [40]. Another mutation with the same functional consequence was already shown in P13, being present in combination with the p.(R485P) mutation and without an ophthalmological phenotype. The exact intronic nucleotides affected by the mutation leading to this deletion are different in both cases (c.373+3G>T versus c.215-1G>T). 

#### 2.4.4. Osseous Phenotype

Eleven patients had facial dysmorphisms and/or a skeletal phenotype (Table 7). Facial dysmorphisms included midfacial hypoplasia with flat nasal bridge and a short nose (P5, P6, P13, P22–P24, P27, and P29). The skeletal phenotype comprised reduced bone mass (P22–24, P27, and P29), chondrodysplasia punctata (P22, and P29), stunted growth (P5, and P6), clinodactyly (P47) and brachytelephalangy of the fingers (P7). Only P22 and P29 had a full osseous phenotype, comprising facial dysmorphia, reduced bone mass and chondrodysplasia punctata. Brachytelephalangy of the fingers is a distinct characteristic of Keutel syndrome (OMIM#245150), an autosomal recessive disorder caused by mutations in *MGP*, a VKDP that is carboxylated by GGCX. After biallelic *GGCX* mutations were identified in this patient, functional analysis showed an abolished carboxylation of MGP, which remained in the inactive state, thus mimicking part of the Keutel syndrome phenotype [28]. The same mimicry also occurred for two other patients with a skeletal phenotype: P13 at birth had facial dysmorphia similar to Williams–Beuren syndrome (OMIM#194050) and P22 had chondrodysplasia punctata similar to Conradi–Hünermann syndrome (X-linked chondrodysplasia punctata; OMIM#302960). None of the patients had causal genetic defects for these syndromes. Apart from the clinodactyly P47 also had ectopic calcification with nephrocalcinosis and mineralization of the trachea and bronchi.

Bone symptoms were associated with nine different genotypes and 13 different mutations, eight of which were located in the HTTM domain (two in TMD1 and -2 and two in TMD3), one mutation affected the N-terminal region and the first part of the HTTM-domain (TMD1), one mutation was localized in the RmlC-like jelly roll fold, one near the C-terminal Gla domain and two near the propeptide binding site. In three patients (P23, P24, and P47), a homozygous genotype was identified: (p.(R204C); p.(R204C)) in P23 and P24 and c.44-1G>A in P47; all other patients had compound heterozygous mutations. The mutation p.(R485P), located near the propeptide binding site (cf. supra), was found twice in compound heterozygous state, respectively, with p.(G72_L124del) and p.(W315X) on the other allele; p.(W157R) was identified in three patients, respectively, with p.(T591K) (P5, P6) and c.2085-5T>C (P28) in *trans*. All patients with an osseous phenotype had at least 1 mutation in the HTTM domain.

#### 2.4.5. VKCFD1

Thirty-three patients had VKCFD1 (Table 8), 25 of which had a deficiency of all VK-dependent coagulation factors (P1–P7, P9, P12–P16, P19–P22, P25, P26, P28–P30, P44, and P47). Defective FX activity was present in all patients, FII impairment in 30 patients, FVII deficiency in 31 patients and FIX was abnormally low in 24 patients. Of note, FIX deficiency was only present in those patients in whom all coagulation factors were deficient. Of the 33 patients with VKCFD1, 21 had symptoms of increased bleeding tendency, including intra-articular bleeding (e.g., knee hemarthrosis), abnormal bleeding after injuries, vaccination or surgery (e.g., after dental extraction) and spontaneous bleeding (e.g., vaginal, cerebral, and gingival). Ten patients were severely affected with symptoms before the age of one year (P1, P2, P7, P15, P16, P23, P26, P30, P44, and P46): all but P23 had a combined deficiency of FII, FVII, FIX and FX; for P46 the FIX function was not determined; and none of the severely affected patients had cutis laxa or other PXE-like skin or eye manifestations. Eleven patients developed a bleeding phenotype at an older age (P5, P6, P10–P12, P17, P19, P24, P25, P29, and P47), four of which had skin symptoms (P10, P11, P17, and P19) and three eye manifestations (P10, P11, and P17) at the time of publication.

Twenty-five VKCFD1 patients received a treatment with VK, the details of which are summarized in Table 9. Regarding the hematological parameters, 17 patients responded well to the treatment (P1, P2, P7, P10, P12 (only international normalized ratio (INR) and aPTT), P13, P15, P21, P22, P23, P24, P27, P28, P29, P44, P45, and P46), five patients showed no or only a limited response (P5, P12 (coagulation factors), P25, P30, and P47) and for three patients the initial VK deficiency was mentioned in the manuscript without details on the individual clotting parameters so an evaluation of the effect of the VK treatment was not possible (P3, P4, and P26). Non-hemostatic parameters were determined before treatment and after initiation of the VK therapy in six patients: in five patients the ratio uncarboxylated(uc) OC/gamma-carboxylated(c) OC decreased significantly but remained supranormal (P21, P27, P28, P29, and P47), in one patient the desphospho-uncarboxylated MGP serum level was measured with no response to treatment (P7). Nine patients had no new bleeding episodes after initiation of the VK treatment (P2, P3, P4, P7, P10, P12, P15, P30, and P46), two patients had recurrent bleeding on VK treatment (P1, and P47) and for six patients the clinical outcome was not mentioned (P5, P14, P21, P23, P24, and P44). In three patients, VKCFD1 was an asymptomatic incidental finding with either no new bleeding after start of VK supplementation (P13, and P22) or no mentioning of the clinical effect of the treatment (P45). In six patients the clinical effect of the VK treatment was mentioned. For five of these patients, a good response to VK supplementation was mentioned without details on the clinical outcome (P25, P26, P27, P28, and P29).

Twenty-one distinct genotypes were associated with VKCFD1, encompassing 33 individual mutations. Fourteen mutations were located in the HTTM domain (one in TMD1, one in TMD2, two in TMD1 and -2, two in TMD3, and one in TMD4), one mutation affected the N-terminal region and the first part of the HTTM domain (TMD1), seven mutations were located in or near the propeptide binding site, two in the glutamate binding site, three in the RmlC-like jelly roll fold, one in the RmlC-like jelly roll fold/near the propeptide binding site, two near the C-terminal Gla-domain, one in TMD5, and one mutation was located in intron 1 of the *GGCX* gene. In the whole cohort of patients with VKCFD1, 14/33 harbored homozygous mutations, whereas in the severely affected cohort 6/9 genotypes were homozygous. Moreover, in the severe cohort, both mutations were located in or near the same domain in all but two patients (P15, and P46). In P17 and P18 only one *GGCX* mutation could be identified. The genotype of 15/33 patients contained no mutations in the HTTM domain. The mutational spectrum between patients with a good or bad response to VK supplementation (clinical and hemostatic parameters) seems similar. With regards to the non-hemostatic parameters, four out of five patients with a decreasing ratio ucOC/cOC harbor at least one mutation in the HTTM domain. There are no patients mentioned who did not have an alteration in this ratio so a potential difference in the mutational spectrum cannot be assessed.

## 3. Discussion

For this systematic review, we assessed all patients described in literature with cardiac, dermatological, ophthalmological, osseous and/or coagulation abnormalities, caused by *GGCX* mutations, and explored possible genotype–phenotype correlations. As the number of patients suffering from these orphan diseases is too small to allow valid statistical interpretation of the data, we did not perform a meta-analysis. However, based on the data, we identified a trend that the presence of at least one *GGCX* mutations in the HTTM domain may predispose for the occurrence of a cardiac and/or osseous phenotype. Further, there seems to be an association between aging and the occurrence of a skin and, less clearly, an ocular phenotype rather than a link with a specific genotype. However, most of the patients with an ophthalmological phenotype also had at least one HTTM domain mutation. Regarding the bleeding phenotype, severely affected patients seem to have homozygous mutations or compound heterozygous mutations affecting the same protein domain.

### 3.1. Cardiac Phenotype

To date, no direct pathophysiological link has been established between *GGCX* mutations and cardiac abnormalities. However, Gas6—a VKDP gamma-carboxylated by GGCX—activates AXL (AXL receptor tyrosine kinase; OMIM*109135), a receptor tyrosine kinase (RTK) that interacts with non-muscle myosin IIB, an essential protein for normal development of the murine heart [51,52]. As all patients with cardiac involvement had at least one mutation located in the HTTM domain, possibly this domain plays a role in Gas6 carboxylation, which could explain the cardiac problems in patients. Cardiac anomalies were found in 8/47 patients (17%) with *GGCX* mutations, in contrast to 4/63 patients (6%) with fetal warfarin syndrome, caused by inhibition of gamma-carboxylation by warfarin administration during pregnancy [53]. A possible explanation for this discrepancy is that warfarin is a dose-dependent inhibitor of the VK cycle and *GGCX* mutations lead to a permanent and persistent defect in gamma-carboxylation [54]. Deficiency of another VKDP, MGP, has also been associated with cardiac involvement, including pulmonary artery stenosis and ventricular septal defects [55,56].

As in the study cohort all patients with cardiac problems have at least one mutation in the HTTM domain, patients with a *GGCX* mutation in this domain might benefit from an echocardiography to rule out possible anomalies or to enable early initiation of treatment if a heart defect is identified. This is for example the case in patients with a small to moderate patent ductus arteriosus Botalli, who may remain asymptomatic during childhood, leaving the defect thus undetected. Some of these patients develop congestive heart failure in early adulthood due to a chronic, left heart overload [57]. Early diagnosis can enable early surgical closure of the patent ductus arteriosus Botalli, hereby preventing the development of heart failure.

### 3.2. Dermatological Phenotype

The patients diagnosed with skin symptoms were often described with a less severe bleeding phenotype, suggesting the presence of a residual carboxylation capacity. Recently, an exon 3 deletion, present in P13 and P31–P43 was proven to completely inactivate the GGCX enzyme. In P31–P43, this mutation (c.373+3G>T), which is localized in TMD1 and -2, was present homozygously and led to cutis laxa, a pigmentary retinopathy but no VKCFD1. Interestingly, P13 harbored a heterozygous exon 3 deletion, caused by another splice site mutation c.215-1G>T, with the p.(R485P) mutation on the other allele and had VKCFD1 but no dermatological phenotype. Jin et al. showed that p.(R485P) led to a GGCX enzyme with some residual function, which according to the authors played an important role in the development of a bleeding phenotype, as a high dose of VK was necessary to ameliorate the phenotype [58]. These data could indicate that TMD1 and -2 have no significant role in the carboxylation of VK-dependent coagulation factors. Indeed, only four patients with VKCFD1 (P13, P15, and P29) have a heterozygous mutation in TMD1 and/or TMD2, and all but one had a second mutation in another GGCX domain. P47 had a homozygous *GGCX* mutation affecting the HTTM (TMD1) and only had VKCFD1 and no skin phenotype. However, the mutation also affected the N-terminal region of the protein, which may be an explanation for the VKCFD1 phenotype in this patient. Further, the absence of a skin phenotype could be due to the young age of the patient when it was described in literature (four years of age at last described follow-up).

Regarding the severity of the skin manifestations, PXE-like dermatological symptoms—mainly cutis laxa—are typically more severe compared to classic PXE patients with regard to their location (beyond flexural areas), number of additional skin folds and the time span during which they develop and aggravate [36]. Interestingly, 21/24 patients discussed in this paper who are older than 18 years, have typical PXE-like skin manifestations (Table 2 and Table 5). This could indicate that skin symptoms may be due to an accumulation of certain substances which only leads to symptoms when a critical threshold is reached, irrespective of the patients’ genotypes. In this respect, Vanakker et al. showed an accumulation of uncarboxylated MGP and OC, two potent mineralization inhibitors, in patients with PXE-like disorder with multiple combined coagulation factor deficiency [36]. Although the age of onset of skin problems is variable between the described patients (P3–P40), this would indicate that most, if not all, patients with biallelic *GGCX* mutations develop skin lesions in the course of their disease. 

For P13, at birth the putative diagnosis of Williams-Beuren syndrome was made based on his facial gestalt. Even though there is no direct link with VKDP and VKCFD1, this disease is caused by a deletion of the WBSCR (Williams–Beuren syndrome critical region), including the *ELN* gene (elastin; OMIM*130160), responsible for the arteriopathy in Williams-Beuren syndrome. Further, cardiac involvement (most commonly supravalvular aortic stenosis) and a skin phenotype (soft loose skin) are features of this disease [59]. Moreover, heterozygous *ELN* mutations are associated with an autosomal dominant type of cutis laxa (ADCL1; OMIM#123700) [60]. These clinical findings might indicate a possible link between these disease entities, although only one patient in the whole cohort was described with a facies resembling the Williams–Beuren syndrome, rendering the assumption less likely.

Of the remaining 24 patients without skin manifestations, 20 were 18 years old or younger and 21/24 had VKFCD1 (deficiency of the VK-dependent coagulation factors (Table 2 and Table 8). Hence, VKFCD1 and the PXE-like disorder with multiple coagulation factor deficiency could belong to a disease spectrum with partly overlapping etiopathogenetic mechanisms, but in whom different GGCX domains are important for the activation of the involved VKDP. However, the young age of these patients could also be an explanation for the absence of a skin phenotype. In severe cases, the additional skin folds may lead to restriction of normal physical activities and may be predilection sites for (severe) skin infections, which is important in patient counseling and follow-up. 

Finally, in the analyzed literature, three patients are described with skin symptoms and a digenic inheritance of *ABCC6* and *GGCX* mutations [37,46]. These patients were not included in this analysis because an unambiguous interpretation of the skin features is not possible, as both *GGCX* and *ABCC6* mutation may lead to related skin phenotypes. Typically, the skin phenotype in these patients is less severe than in patients with biallelic *GGCX* mutations, so it may be worthwhile to perform additional sequencing of the *ABCC6* gene in those patients in whom only one *GGCX* mutation is withheld as it will influence genetic counseling and management.

### 3.3. Ophthalmological Phenotype

Regarding the eye symptoms, all patients with a homozygous skip of exon 3 were diagnosed with a pigmentary retinopathy, a disease mimicking retinitis pigmentosa. In mice, it has already been shown that absence of both ligands of the RTK MerTK (Mer tyrosine kinase proto-oncogene; OMIM*604705), Gas6 and ProS, leads to retinitis pigmentosa [61]. In analogy, such a mechanism could play a role in the development of a pigmentary retinopathy in the two families with a homozygous skip of exon 3 in the *GGCX* gene. As this mutation is located in the HTTM domain, this could again point to its putative roles in the carboxylation of both Gas6 and ProS, similar to what is observed in patients with a cardiac phenotype. 

Furthermore, as in 17 of the 24 patients over 18 years of age an ophthalmological phenotype was identified and in only four out of 23 patients with cutis laxa no accompanying eye disease was reported, possibly, the major determinant of developing a GGCX-related retinopathy is also increasing age. Although this link seems less convincing compared to skin manifestations, it should be noted that (mild) angioid streaks are asymptomatic and can easily be overlooked during a routine ophthalmological checkup. In the spectrum, PXE-like skin and eye symptoms may be phenotypes that occur in most patients with increasing age, whereas VKCFD1 may be more variable, with neonatal complications in severe forms to apparent normal coagulation in very mildly affected patients, such as those patients described by Kariminejad et al. [40] with PXE-like skin symptoms, an eye phenotype but no apparent VKCFD1. As mentioned above, possibly distinct GGCX domains play an important part role in the gamma-carboxylation of involved VKDP.

Overall, the PXE-like ophthalmological symptoms (P8, P10, P11, P16, P17, and P18) seem less severe compared to the dermatological features with no functional complications in the described patient cohort. However, as this cohort is small, we cannot exclude the possibility that in some patients with biallelic *GGCX* mutations more severe PXE eye symptoms may occur with subretinal neovascularization and hence loss of vision if not treated immediately. Further, a pigmentary retinopathy with severe visual dysfunction may occur in some patients. It therefore seems appropriate to follow patients ophthalmologically, certainly from the time they start to develop skin symptoms. In mildly affected patients the typical PXE-like angioid streaks may be very small and thus only be picked up by very sensitive funduscopic imaging, for which confocal near-infrared reflectance imaging is superior compared to other techniques [62,63]. If symptoms are stable for a long time, follow-up may become less stringent; however, to our knowledge, there has thus far not been a comprehensive long-term follow-up study of eye symptoms in patients with biallelic *GGCX* mutations.

### 3.4. Osseous Phenotype

Bone symptoms in patients with *GGCX* mutations show an important clinical overlap with other genetic syndromes, such as X-linked chondrodysplasia punctata (an autosomal recessive disorder caused by mutations in the *ARSE* gene (arylsulphatase E; OMIM*123700), encoding ARSE), Keutel syndrome, due to *MGP* gene mutations; and the fetal warfarin syndrome [64]. Interestingly, Vanakker et al. confirmed an accumulation of uncarboxylated MGP and OC in patients with the PXE-like syndrome; similarly, an in vitro model for the mutation leading to the Keutel syndrome-like phenotype in P7 showed an abolishment of MGP carboxylation [28,36]. For ARSE, no data are available of a direct association with GGCX. However, it was shown that ARSE is specifically inhibited by warfarin, therefore having a possible role in warfarin embryopathy, thus ARSE could have a role in the VK cycle, which would explain the phenotypic overlap with patients with biallelic *GGCX* mutations [65]. Further investigation of this interaction proves a valid way to further unravel the association between the VK-dependent pathway and skeletal development. 

Finally, all patients with skeletal and/or facial symptoms had at least one *GGCX* mutation in the HTTM-domain, so putatively this domain not only plays a role in Gas6 and ProS carboxylation but also in MGP (and OC) carboxylation. Because of the association with osteopenia and osteoporosis, patients with a mutation in the HTTM domain may benefit from an early densitometry (as P23 had already developed osteoporosis at 12 years old, first densitometry may be most valuable at a younger age) to facilitate early treatment when a decreased bone density is present. There is no genotypic difference between patients with a complete bone phenotype (chondrodysplasia punctata, reduced bone mass, facial dysmorphisms) or with only some of the osseous features. Therefore, early bone densitometry may better be advised to all patients with at least one mutation in the HTTM domain.

### 3.5. VKCFD1

Overall, VKCFD1 seems to be variable in severity, clinical presentation and response to VK supplementation in the analyzed patient cohort. However, there seems to be a trend that the genotypes of patients with very early onset of bleeding symptoms (<1 year of age) mainly comprise homozygous mutations or compound heterozygous mutations in or near the same protein domain. Possibly, biallelic hits in the same protein domain lead to a more severe malfunctioning of the carboxylase and thus more severe VKCFD1 symptoms occur in these patients at a younger age. However, this could not be confirmed by the activity of the coagulation factors in these patients, even though it is difficult to compare such values between patients whose coagulation factor function is measured in different labs, as different units and reference values are used, which cannot easily be compared to each other. 

None of the patients with severe bleeding symptoms had a skin or eye phenotype. However, this could be a bias, as these severely affected patients are often described at a very young age and no follow-up studies are available, so putative development of cutis laxa or other PXE-like skin and/or eye manifestations at a later age remains a possibility, which is exemplified by the presence of skin symptoms in some of the patients who have a rather mild bleeding phenotype. 

In a small subset of the patient cohort carboxylation status of extrahepatic VKDP were measured, and OC carboxylation seemed to respond (to a variable extent) to VK supplementation. Interestingly, in one of these patients, VK treatment did not influence the bleeding phenotype with no significant change in hemostatic parameters and recurrent bleeding episodes after initiation of the treatment. In contrast, another patient showed a distinct amelioration of the coagulation factor function on VK treatment, whereas MGP carboxylation did not increase. This variability in response to VK supplementation may result from different mechanisms in gamma-carboxylation of hemostatic and extrahepatic VKDP [28]. Moreover, different *GGCX* mutations affect different regions of the GGCX protein, which could interfere with propeptide binding of different VKDP [21]. The cohort is however too small to make definite conclusions.

## 4. Materials and Methods 

### 4.1. Article Selection and Patient Data Extraction

For this review, Pubmed was systematically searched for all papers about GGCX-related phenotypes on 14 December 2016, using the following key words: “GGCX OR gamma-glutamyl carboxylase OR Vitamin K-dependent carboxylase OR γ-carboxylase AND mutation”. The inclusion criteria were as follows: all article types were included (case reports and series, systematic reviews, original articles), as the data about the subject is limited; neither the date of publication nor the journal played a role in the selection; only articles in English, French or Dutch were considered; only articles of which the full text was available were included in the analysis; patients should have at least 1 relevant GGCX-related symptom (cardiac, dermatological, ophthalmological, osseous or coagulation feature) with no limitations regarding age, ethnicity or symptoms. 

Only articles meeting the inclusion criteria were included in this systematic review. For each patient, patient characteristics (age, sex, nationality or ethnicity, age of first skin symptoms), genotype and the GGCX-related phenotypes (cardiac, dermatological, ophthalmological, osseous and coagulation dysfunction) were extracted from the original article. For the bleeding phenotype, biochemical values of the VK-dependent coagulation factors (FII, FVII, FIX and FX), PT, INR, aPTT an extrahepatic VKDP (if mentioned) were collected. Coagulation factor function was abnormal when they were not within the normal range of the reference value given in the original paper or, if no reference values were mentioned in the article, when it was clearly stated in the report that the coagulation factor function was deficient. Patients with bleeding symptoms under the age of 1 year were defined as severely affected VKCFD1 patients. Patients were only classified in the severe group when the exact age was clearly mentioned in the original article.

### 4.2. Genotype Analysis

Mutations were annotated according to the Human Genome Variation Society (HGVS) recommendations at the cDNA and protein level. Further, for each mutation the corresponding protein domain was mentioned if possible (Table 2). Reference sequence NM000821.6 (ENST00000233838.8) of the *GGCX* gene was used to verify the annotation of all mutations at the cDNA and protein level, as this represents the longest transcript and encodes the longest GGCX isoform. If mutations were annotated incorrectly by using a different reference sequence or another unspecified sequence, the annotation was updated using reference sequence NM000821.6, in order to enable comparison of all genotypes and to establish genotype–phenotype correlations in the complete patient cohort. 

## 5. Conclusions

Gamma-carboxylation is an essential process in the activation of VKDP, which are important in numerous biological processes, such as blood clotting, inflammation, bone formation and cell proliferation. This posttranslational modification process is executed by the GGCX enzyme. Mutations in the gene encoding GGCX have been linked to multiple distinct phenotypes, affecting the heart, skin, eyes, blood clotting and bone metabolism. This review highlights the importance of mutations in the HTTM domain for at least the cardiac and bone phenotype, as all of the patients had at least one mutation in this domain, whereas multiple patients without cardiac or osseous manifestations had no mutations in the HTTM domain. Further, age was identified as the most important determinant of the development of PXE-like skin symptoms and to a lesser extent ophthalmological manifestations. Finally, distinct parts of the HTTM domain seem to have a specific role in the development of skin symptoms and not of VKCFD1. Based on our results, patients should be informed during genetic counseling about the possibility of skin lesions appearing in the course of their disease, taking into account that these lesions may be subtle at onset. In all, a detailed ophthalmological evaluation should be performed and adequate follow-up should be organized, as apart from a (mild) typical PXE-like retinopathy a pigmentary retinopathy with significant functional implications may also occur. Because of its association with reduced bone mass, a bone densitometry should be offered to all patients harboring at least one mutation in the HTTM domain of GGCX.

The main limitations of this systematic review are the low number of patients with GGCX-related phenotypes and the presence of consanguinity in some of the assessed families. There is a possibility that homozygosity at other loci could play a role in the occurrence of the other GGCX-related phenotypes in these patients. However, for most of these phenotypes, there are clinical discrepancies between siblings, which makes this less suggestive, even though the possibility of variable penetrance cannot be ruled out. These limitations are inherent to this type of autosomal recessive disorders, because most of them are rare hence making it impossible to have large cohorts with only individual probands. Another limitation is the incomplete information regarding the (non-)hemostatic parameters in some of the original papers, which do not mention reference values, making it impossible to correctly interpret the values in comparison to other patients, as the reference values tend to be highly laboratory-dependent. Further, the details of the VK treatment, with regards to mode of administration, VK dose, specific subtype of VK, and duration of the therapy, are often not or incompletely mentioned. These details could possibly influence the response to treatment in the described patients. However, the main purpose of this systematic review was to combine all known and described patients with GGCX-related phenotypes and to explore the possibility of genotype–phenotype correlations for the different phenotypes. This manuscript can be used as a guideline for future research on the GGCX protein structure and function, which then can lead to new insights in the VK cycle.

## Figures and Tables

**Table 1:** Overview of the phenotypic features of the known gamma-glutamyl carboxylase (GGCX)-related disease entities.

Disease	Coagulation Deficit	Bone	Cardiac	Skin	Ocular	Other
VKCFD1	yes	If present: midfacial hypoplasia; reduced bone mass; chondrodysplasia; punctate (most frequent)	If present: PDA; SCD (most frequent)	no	no	no
PXE-like	yes	no	no	yellowish skin papules; skin elasticity loss; skin loosening (resembling cutis laxa)	peau d’orange; angioid streaks	no
PXE/PXE-like	no	no	no	reddish rash; excessive skin folds; mild yellowish rash	peau d’orange; angioid streaks	abdominal calcifications
PXE + PR	no	no	no	yellowish skin papules; loss of skin elasticity; skin loosening (resembling cutis laxa)	pigmentary retinopathy: rod responses non-detectable or reduced amplitude; prolonged implicit time	no

PDA: persistent ductus arteriosus Botalli; PR: pigmentary retinopathy; PXE: pseudoxanthoma elasticum; PXE-like: PXE-like disorder with combined coagulation factor deficiency; SCD: septal closure defects; VKCFD1: vitamin K-dependent clotting factor deficiency-1.

**Table 2:** Patient characteristics. This table gives an overview of all the patients included in this systematic review. For each patient the GGCX mutations are mentioned (cDNA and protein annotation) as well as the protein domain in which the affected amino acid is situated. Further, for each patient, the age, nationality/ethnicity and sex are provided.

Id	Original Id	Allele 1			Allele 2			Age *	Nationality/Ethnicity	Sex	References
		c.Annotation	p.Annotation	Protein Domain	c.Annotation	p.Annotation	Protein Domain				
1	no. 20	c.1181T>G	p.(L394R)	Glu-BS	c.1181T>G	p.(L394R)	Glu-BS	NP/14	Arabic	F	Brenner et al., 1998 [13]
2	no. 21	c.1181T>G	p.(L394R)	Glu-BS	c.1181T>G	p.(L394R)	Glu-BS	5m/7	Arabic	F	Brenner et al., 1998 [13]
3	no. 22	c.1181T>G	p.(L394R)	Glu-BS	c.1181T>G	p.(L394R)	Glu-BS	NP/30	Arabic	M	Brenner et al., 1998 [13]
4	no. 23	c.1181T>G	p.(L394R)	Glu-BS	c.1181T>G	p.(L394R)	Glu-BS	NP/30	Arabic	F	Brenner et al., 1998 [13]
5	proposita	c.469T>C	p.(W157R)	HTTM (TMD3)	c.1772C>A	p.(T591K)	RmlC-like	2/11	Tunisian	F	Dargouth et al., 2006 [24]
6	brothers	c.469T>C	p.(W157R)	HTTM (TMD3)	c.1772C>A	p.(T591K)	RmlC-like	1	Tunisian	M	Dargouth et al., 2006 [24]
7	patient	c.458A>G	p.(D153G)	HTTM (TMD3)	c.521T>G	p.(M174R)	HTTM	4m	nd	F	Tie et al., 2016 [28]
8	III-3	c.763G>A	p.(V255M)	HTTM	c.899C>T	p.(S300F)	HTTM (TMD4)	16	Caucasian	F	Li et al., 2009 [37]
9	III-1	c.763G>A	p.(V255M)	HTTM	c.899C>T	p.(S300F)	HTTM (TMD4)	19	Caucasian	F	Li et al., 2009 [37]
10	A.B. [39], II-2 [40]	c.274C>T	p.(R83W)	HTTM	c.1120C>T	p.(Q374X)	TMD5	13/48	Caucasian	F	Goldsmith et al., 1982 [47], Li et al., 2009 [48]
11	M.F. [39], II-3 [40]	c.274C>T	p.(R83W)	HTTM	c.1120C>T	p.(Q374X)	TMD5	18/46	Caucasian	M	Goldsmith et al., 1982 [47], Li et al., 2009 [48]
12	patient	c.521T>G	p.(M174R)	HTTM	c.1595T>C	p.(I532T)	RmlC-like	18	Italian	M	Lunghi et al., 2011 [26]
13	A	c.215-1G>T	p.(G72_L124del)	HTTM (TMD1 and -2)	c.1454G>C	p.(R485P)	near PP-BS	1	German	M	Rost et al., 2004 [23], Rost et al., 2006 [49]
14	pt 4	c.1502G>C	p.(W501S)	PP-BS	c.1502G>C	p.(W501S)	PP-BS	7d	Lebanese	M	Spronk et al., 2000 [22]
15	patient	c.214+1G>T	Splice	HTTM (TMD1)	c.1609+3A>G	Splice	near PP-BS + RmlC-like	birth/6	Mexican	M	Titapiwatanakun et al., 2009 [25]
16	pt 1	c.1478G>C	p.(W493S)	near PP-BS	c.1478G>C	p.(W493S)	near PP-BS	46	Caucasian	F	Vanakker et al., 2007 [36], Watzka et al., 2014 [21]
17	pt 3	c.1426C>T	p.(R476C)	near PP-BS	-	-	-	67	Caucasian	F	Vanakker et al., 2007 [36], Watzka et al., 2014 [21]
18	pt 4	c.1427G>A	p.(R476H)	near PP-BS	-	-	-	32	Caucasian	F	Vanakker et al., 2007 [36], Watzka et al., 2014 [21]
19	pt 5	c.1120C>T	p.(Q374X)	TMD5	c.1610G>C	p.(G537A)	RmlC-like	46	Caucasian	F	Vanakker et al., 2007 [36], Watzka et al., 2014 [21]
20	pt 6	c.1120C>T	p.(Q374X)	TMD5	c.1610G>C	p.(G537A)	RmlC-like	44	Caucasian	M	Vanakker et al., 2007 [36], Watzka et al., 2014 [21]
21	B [41], A [19]	c.1211A>C	p.(H404P)	Glu-BS	c.1454G>C	p.(R485P)	near PP-BS	22/38	German	F	Rost et al., 2006 [49], Watzka et al., 2014 [21]
22	B	c.944G>A	p.(W315X)	HTTM	c.1454G>C	p.(R485P)	near PP-BS	14/20	German	F	Watzka et al., 2014 [21]
23	C1	c.610C>T	p.(R204C)	HTTM	c.610C>T	p.(R204C)	HTTM	2d/11	former Yugoslavia	F	Watzka et al., 2014 [21]
24	C2	c.610C>T	p.(R204C)	HTTM	c.610C>T	p.(R204C)	HTTM	10/14	former Yugoslavia	F	Watzka et al., 2014 [21]
25	D	c.248G>T	p.(R83P)	HTTM	c.248G>T	p.(R83P)	HTTM	1/3	Turkish	F	Watzka et al., 2014 [21]
26	E	c.610C>T	p.(R204C)	HTTM	c.610C>T	p.(R204C)	HTTM	2m/4	Turkish	M	Watzka et al., 2014 [21]
27	F	c.469T>C	p.(W157R)	HTTM (TMD3)	c.2085-5T>C	Splice	near Gla domain	12/14	Italian	F	Watzka et al., 2014 [21]]
28	G	c.850T>C	p.(S284P)	HTTM	c.944G>A	p.(W315X)	HTTM	11/13	German	F	Watzka et al., 2014 [21]
29	H	c.373G>A	p.(G125R)	HTTM (TMD2)	c.1601A>T	p.(D534V)	near PP-BS+RmlC-like	5m/5	German-Tunisian	M	Watzka et al., 2014 [21]
30	patient	c.1502G>C	p.(W501S)	PP-BS	c.1502G>C	p.(W501S)	PP-BS	3d/3,5	Lebanese	F	Moussalem et al., 2001 [50]
31	V4	c.373+3G>T	p.(F74_G125del)	HTTM (TMD1 and -2)	c.373+3G>T	p.(F74_G125del)	HTTM (TMD1 and -2)	11/40	Iranian	F	Kariminejad et al., 2014 [40]
32	V6	c.373+3G>T	p.(F74_G125del)	HTTM (TMD1 and -2)	c.373+3G>T	p.(F74_G125del)	HTTM (TMD1 and -2)	12/52	Iranian	M	Kariminejad et al., 2014 [40]
33	V7	c.373+3G>T	p.(F74_G125del)	HTTM (TMD1 and -2)	c.373+3G>T	p.(F74_G125del)	HTTM (TMD1 and -2)	12/37	Iranian	M	Kariminejad et al., 2014 [40]
34	V10	c.373+3G>T	p.(F74_G125del)	HTTM (TMD1 and -2)	c.373+3G>T	p.(F74_G125del)	HTTM (TMD1 and -2)	25/48	Iranian	F	Kariminejad et al., 2014 [40]
35	V11	c.373+3G>T	p.(F74_G125del)	HTTM (TMD1 and -2)	c.373+3G>T	p.(F74_G125del)	HTTM (TMD1 and -2)	14/40	Iranian	F	Kariminejad et al., 2014 [40]
36	V12	c.373+3G>T	p.(F74_G125del)	HTTM (TMD1 and -2)	c.373+3G>T	p.(F74_G125del)	HTTM (TMD1 and -2)	12/34	Iranian	M	Kariminejad et al., 2014 [40]
37	V14	c.373+3G>T	p.(F74_G125del)	HTTM (TMD1 and -2)	c.373+3G>T	p.(F74_G125del)	HTTM (TMD1 and -2)	14/16	Iranian	F	Kariminejad et al., 2014 [40]
38	V16	c.373+3G>T	p.(F74_G125del)	HTTM (TMD1 and -2)	c.373+3G>T	p.(F74_G125del)	HTTM (TMD1 and -2)	10/21	Iranian	M	Kariminejad et al., 2014 [40]
39	V17	c.373+3G>T	p.(F74_G125del)	HTTM (TMD1 and -2)	c.373+3G>T	p.(F74_G125del)	HTTM (TMD1 and -2)	16/23	Iranian	M	Kariminejad et al., 2014 [40]
40	V18	c.373+3G>T	p.(F74_G125del)	HTTM (TMD1 and -2)	c.373+3G>T	p.(F74_G125del)	HTTM (TMD1 and -2)	11/29	Iranian	M	Kariminejad et al., 2014 [40]
41	IV1	c.373+3G>T	p.(F74_G125del)	HTTM (TMD1 and -2)	c.373+3G>T	p.(F74_G125del)	HTTM (TMD1 and -2)	14/21	Iranian	M	Kariminejad et al., 2014 [40]
42	IV5	c.373+3G>T	p.(F74_G125del)	HTTM (TMD1 and -2)	c.373+3G>T	p.(F74_G125del)	HTTM (TMD1 and -2)	12/28	Iranian	M	Kariminejad et al., 2014 [40]
43	IV6	c.373+3G>T	p.(F74_G125del)	HTTM (TMD1 and -2)	c.373+3G>T	p.(F74_G125del)	HTTM (TMD1 and -2)	12/21	Iranian	F	Kariminejad et al., 2014 [40]
44	A.K.	14 bp del I1	-	-	14 bp del I1	-	-	3m/9	-	M	Thomas et al., 2003 [27]
45	D.K.	14 bp del I1	-	-	14 bp del I1	-	-	6/15	-	F	Thomas et al., 2003 [27]
46	propositus	c.1479G>T	p.(W493C)	Near PP-BS	c.2110C>T	p.(R704X)	Near Gla-domain	Birth/1	French	M	Darghouth et al., 2009 [29]
47	infant	c.44-1G>A	p.(D15_F71del)	N-terminus + HTTM (TMD1)	c.44-1G>A	p.(D15_F71del)	N-terminus + HTTM (TMD1)	14m/4	Caucasian	M	Dasi et al., 2016 [30]

C.annotation: cDNA-annotation; p.annotation: protein annotation; d: days; m: months (in “Age” column only); F: female (in “Sex” column only); Gla: gamma-carboxyglutamate; Glu-BS: glutamate binding site; HTTM: horizontally transferred transmembrane domain; Id: identification number; M: male (in “Sex” column only); m: month(s) (in “Age” column only); NP: neonatal period; PP-BS: propeptide binding site; pt: patient; RmlC: deoxythymidine-6-deoxy-d-xylo-4-hexulose 3,5 epimerase (EC5.1.3.13); RmlC-like: RmlC-like jelly roll fold; splice: splice site mutation; TMD: transmembrane domain; 14 bp del I1: 14 base pair deletion in intron 1 of the *GGCX* gene; * age is stated in years, unless otherwise specified; notation: age of first symptoms or of first examination/age a last follow-up.

**Table 3:** Update of the mutation annotation according to reference sequence NM00082. In the original articles, reference sequence BC013979 was used (+1 located at the −28 position of NM000821).

cDNA Annotation in Original Article	Corrected cDNA Annotation	Protein Annotation	Original Article
c.791G>A	c.763G>A	p.(V255M)	Li et al., 2009 [37]
c.927C>T	c.899C>T	p.(S300F)	Li et al., 2009 [37]
c.1148C>T	c.1120C>T	p.(Q374X)	Li et al., 2009 [48]
c.1454G>C	c.1426C>T	p.(R476C)	Vanakker et al., 2007 [36]
c.1455G>A	c.1427G>A	p.(R476H)	Vanakker et al., 2007 [36]
c.1149C>T	c.1120C>T	p.(Q374X)	Vanakker et al., 2007 [36]
c.1339G>T	c.1610G>C	p.(G537A)	Vanakker et al., 2007 [36]
c.1530G>C	c.1502G>C	p.(W501S)	Spronk et al., 2000 [22], Moussalem et al., 2001 [50]
c.1358+1G>T	c.214+1G>T	Splice site mutation	Titapiwatanakun et al., 2009 [25]
c.10364+3A>G	c.1609+3A>G	Splice site mutation	Titapiwatanakun et al., 2009 [25]
c.1565G>T	c.1565G>T	p.(W493C)	Darghouth et al., 2009 [29]
c.2196C>T	c.2100C>T	p.(R704X)	Darghouth et al., 2009 [29]

For Darghouth et al., 2009 and Titapiwatanakun et al., 2009 the reference sequence was not mentioned.

**Table 4:** Cardiac involvement in patients with *GGCX* mutations. This table gives an overview of all patients in the analyzed cohort with cardiac involvement. For each patient, the *GGCX* mutations on both alleles and the affected protein domain are stated (protein annotation). Further, a brief overview of the cardiac symptoms is shown.

Id	Allele 1		Allele 2		SCD	PDA	Other
	Annotation	Protein Domain	Annotation	Protein Domain			
5	p.(W157R)	HTTM (TMD3)	p.(T591K)	RmlC-like	x		
6	p.(W157R)	HTTM (TMD3)	p.(T591K)	RmlC-like	x		
8	p.(V255M)	HTTM	p.(S300F)	HTTM (TMD4)			*
13	p.(G72_L124del)	HTTM (TMD1 and -2)	p.(R485P)	near PP-BS		x	
25	p.(R83P)	HTTM	p.(R83P)	HTTM	x		
27	p.(W157R)	HTTM (TMD3)	c.2085-5T>C (splice)	near Gla domain		x	
28	p.(S284P)	HTTM	p.(W315X)	HTTM	x		
47	p.(D15_F71del)	N-terminus + HTTM (TMD1)	p.(D15_F71del)	N-terminus + HTTM (TMD1)	x		^†^

Gla: gamma-carboxyglutamate; HTTM: horizontally transferred transmembrane domain; Id: identification number; PDA: patent ductus arteriosus Botalli; PP-BS: propeptide binding site; RmlC: deoxythymidine-6-deoxy-d-xylo-4-hexulose 3,5 epimerase (EC5.1.3.13); RmlC-like: RmlC-like jelly roll fold; SCD: septal closure defects; splice: splice site mutation; TMD: transmembrane domain; * congenital supravalvular pulmonary stenosis and peripheral pulmonary artery stenosis; ^†^ Wolff-Parkinson-White syndrome.

**Table 5:** GGCX-related skin manifestations in analyzed patient cohort. This table gives an overview of all patients in the analyzed cohort with skin features. For each patient, the *GGCX* mutations on both alleles and the affected protein domain are stated (protein annotation). Further, a brief overview of the skin symptoms is shown.

Id	Allele 1		Allele 2		CL	YP	Age of Onset (years)
	Annotation	Protein Domain	Annotation	Protein Domain			
8	p.(V255M)	HTTM	p.(S300F)	HTTM (TMD4)	x		10
9	p.(V255M)	HTTM	p.(S300F)	HTTM (TMD4)	x		early teens
10	p.(R83W)	HTTM	p.(Q374X)	TMD5	x	x	27
11	p.(R83W)	HTTM	p.(Q374X)	TMD5	x	x	nd
16	p.(W493S)	near PP-BS	p.(W493S)	near PP-BS	x		18
17	p.(R476C)	near PP-BS	-	-	x	x	3
18	p.(R476H)	near PP-BS	-	-	x	x	18
19	p.(Q374X)	TMD5	p.(G537A)	Rmlc-like	x	x	-
20	p.(Q374X)	TMD5	p.(G537A)	Rmlc-like	x	x	-
21	p.(H404P)	Glu-BS	p.(R485P)	near PP-BS	x	x	puberty
31	p.(F74_G125del)	HTTM (TMD1 and -2)	p.(F74_G125del)	HTTM (TMD1 and -2)	x	x	11
32	p.(F74_G125del)	HTTM (TMD1 and -2)	p.(F74_G125del)	HTTM (TMD1 and -2)	x	x	12
33	p.(F74_G125del)	HTTM (TMD1 and -2)	p.(F74_G125del)	HTTM (TMD1 and -2)	x	x	12
34	p.(F74_G125del)	HTTM (TMD1 and -2)	p.(F74_G125del)	HTTM (TMD1 and -2)	x	x	25
35	p.(F74_G125del)	HTTM (TMD1 and -2)	p.(F74_G125del)	HTTM (TMD1 and -2)	x	x	14
36	p.(F74_G125del)	HTTM (TMD1 and -2)	p.(F74_G125del)	HTTM (TMD1 and -2)	x	x	12
37	p.(F74_G125del)	HTTM (TMD1 and -2)	p.(F74_G125del)	HTTM (TMD1 and -2)	x	x	14
38	p.(F74_G125del)	HTTM (TMD1 and -2)	p.(F74_G125del)	HTTM (TMD1 and -2)	x	x	10
39	p.(F74_G125del)	HTTM (TMD1 and -2)	p.(F74_G125del)	HTTM (TMD1 and -2)	x	x	16
40	p.(F74_G125del)	HTTM (TMD1 and -2)	p.(F74_G125del)	HTTM (TMD1 and -2)	x	x	11
41	p.(F74_G125del)	HTTM (TMD1 and -2)	p.(F74_G125del)	HTTM (TMD1 and -2)	x	x	14
42	p.(F74_G125del)	HTTM (TMD1 and -2)	p.(F74_G125del)	HTTM (TMD1 and -2)	x	x	12
43	p.(F74_G125del)	HTTM (TMD1 and -2)	p.(F74_G125del)	HTTM (TMD1 and -2)	x	x	12

CL: cutis laxa; Glu-BS: glutamate binding site; HTTM: horizontally transferred transmembrane domain; Id: identification number; PP-BS: propeptide binding site; RmlC: deoxythymidine-6-deoxy-d-xylo-4-hexulose 3,5 epimerase (EC5.1.3.13); RmlC-like: RmlC-like jelly roll fold; TMD: transmembrane domain; YP: yellow papules.

**Table 6:** Eye phenotype in patients with *GGCX* mutations. This table gives an overview of all patients in the analyzed cohort with ophthalmological manifestations. For each patient, the *GGCX* mutations on both alleles and the affected protein domain are stated (protein annotation). Further, a brief overview of the eye symptoms is shown.

Id	Allele 1		Allele 2		AS	Pd’O	PR
	Annotation	Protein Domain	Annotation	Protein Domain			
8	p.(V255M)	HTTM	p.(S300F)	HTTM (TMD4)			
10	p.(R83W)	HTTM	p.(Q374X)	TMD5	x		
11	p.(R83W)	HTTM	p.(Q374X)	TMD5	x	x	
16	p.(W493S)	near PP-BS	p.(W493S)	near PP-BS	x		
17	p.(R476C)	near PP-BS	-	-	x		
18	p.(R476H)	near PP-BS	-	-	x	x	
31	p.(F74_G125del)	HTTM (TMD1 and -2)	p.(F74_G125del)	HTTM (TMD1 and -2)			x
32	p.(F74_G125del)	HTTM (TMD1 and -2)	p.(F74_G125del)	HTTM (TMD1 and -2)			x
33	p.(F74_G125del)	HTTM (TMD1 and -2)	p.(F74_G125del)	HTTM (TMD1 and -2)			x
34	p.(F74_G125del)	HTTM (TMD1 and -2)	p.(F74_G125del)	HTTM (TMD1 and -2)			x
35	p.(F74_G125del)	HTTM (TMD1 and -2)	p.(F74_G125del)	HTTM (TMD1 and -2)			x
36	p.(F74_G125del)	HTTM (TMD1 and -2)	p.(F74_G125del)	HTTM (TMD1 and -2)			x
37	p.(F74_G125del)	HTTM (TMD1 and -2)	p.(F74_G125del)	HTTM (TMD1 and -2)			x
38	p.(F74_G125del)	HTTM (TMD1 and -2)	p.(F74_G125del)	HTTM (TMD1 and -2)			x
39	p.(F74_G125del)	HTTM (TMD1 and -2)	p.(F74_G125del)	HTTM (TMD1 and -2)			x
40	p.(F74_G125del)	HTTM (TMD1 and -2)	p.(F74_G125del)	HTTM (TMD1 and -2)			x
41	p.(F74_G125del)	HTTM (TMD1 and -2)	p.(F74_G125del)	HTTM (TMD1 and -2)			x
42	p.(F74_G125del)	HTTM (TMD1 and -2)	p.(F74_G125del)	HTTM (TMD1 and -2)			x
43	p.(F74_G125del)	HTTM (TMD1 and -2)	p.(F74_G125del)	HTTM (TMD1 and -2)			x

AS: angioid streaks; HTTM: horizontally transferred transmembrane domain; Id: identification number; Pd’O: peau d’orange; PP-BS: propeptide binding site; PR: pigmentary retinopathy; TMD: transmembrane domain.

**Table 7:** Osseous involvement in patients with *GGCX* mutations. This table gives an overview of all patients in the analyzed cohort with osseous manifestations features. For each patient, the *GGCX* mutations on both alleles and the affected protein domain are stated (protein annotation). Further, a brief overview of the bone features is shown.

Id	Allele 1		Allele 2		FD	CP	RBM	Other
	Annotation	Protein Domain	Annotation	Protein Domain				
5	p.(W157R)	HTTM (TMD3)	p.(T591K)	RmlC-like	x			*
6	p.(W157R)	HTTM (TMD3)	p.(T591K)	RmlC-like				^†^
7	p.(D153G)	HTTM (TMD3)	p.(M174R)	HTTM				^‡^
13	p.(G72_L124del)	HTTM (TMD1 and -2)	p.(R485P)	near PP-BS	x			
22	p.(W315X)	HTTM	p.(R485P)	near PP-BS	x	x	x	
23	p.(R204C)	HTTM	p.(R204C)	HTTM	x		x	
24	p.(R204C)	HTTM	p.(R204C)	HTTM			x	
27	p.(W157R)	HTTM (TMD3)	c.2085-5T>C (splice)	Gla domain	x	x	x	
28	p.(S284P)	HTTM	p.(W315X)	HTTM	x			
29	p.(G125R)	HTTM (TMD2)	p.(D534V)	near PP-BS+RmlC-like	x		x	
47	p.(D15_F71del)	N-terminus + HTTM (TMD1)	p.(D15_F71)del	N-terminus + HTTM (TMD1)				^§^

CP: chondrodysplasia punctata; FD: facial dysmorphia; Gla: gamma-carboxyglutamate; HTTM: horizontally transferred transmembrane domain; Id: identification number; PP-BS: propeptide binding site; RBM: reduced bone mass; RmlC: deoxythymidine-6-deoxy-d-xylo-4-hexulose 3,5 epimerase (EC5.1.3.13); RmlC-like: RmlC-like jelly roll fold; splice: splice site mutation; RmlC-like: RmlC-like jelly roll fold; TMD: transmembrane domain; * stunted growth, ^†^ skeletal abnormalities, ^‡^ telebrachydactyly, ^§^ clinodactyly, nephrocalcinosis and calcification of the trachea and bronchi.

**Table 8:** VKCFD1 in patients with *GGCX* mutations. This table gives an overview of all patients in the analyzed cohort with VKCFD1. For each patient, the *GGCX* mutations on both alleles and the affected protein domain are stated (protein annotation). Further, an overview of the coagulation factor function (percent of normal activity or in U/dL or U/mL), aPTT and PT is given, it is stated if patients were symptomatic within the first year of life. Reference values are stated between brackets if they were mentioned in the original article.

Id	Allele 1		Allele 2		PT	INR	aPTT	FII	FVII	FIX	FX	Symptoms in 1st Year of Life
	Annotation	Protein Domain	Annotation	Protein Domain								
1	p.(L394R)	Glu-BS	p.(L394R)	Glu-BS	>120 s	-	>180 s	2 U/dL [77–125 U/dL]	3 U/dL [63–139 U/dL]	8 U/dL [63–155 U/dL]	2 U/dL [55–160 U/dL]	yes
2	p.(L394R)	Glu-BS	p.(L394R)	Glu-BS	-	-	-	24 U/dL [77–125 U/dL]	23 U/dL [63–139 U/dL]	8 U/dL [63–155 U/dL]	20 U/dL [55–160 U/dL]	yes
3	p.(L394R)	Glu-BS	p.(L394R)	Glu-BS	nd *	nd *	nd *	nd *	nd *	nd *	nd *	no
4	p.(L394R)	Glu-BS	p.(L394R)	Glu-BS	nd *	nd *	nd *	nd *	nd *	nd *	nd *	no
5	p.(W157R)	HTTM (TMD3)	p.(T591K)	RmlC-like	49.5 s [11.5 s]	-	60 s [30 s]	9% *	6% *	7% *	5% *	no
6	p.(W157R)	HTTM (TMD3)	p.(T591K)	RmlC-like	>60 s [11.5 s]	-	56 s [30 s]	14% *	7% *	nd *	7% *	no
7	p.(D153G)	HTTM (TMD3)	p.(M174R)	HTTM	11% [70%–100%]	-	ratio 1.65 [0.84–1.21]	26% [50%–150%]	<1% [50%–150%]	12% [50%–150%]	13% [50%–150%]	yes
8	p.(V255M)	HTTM	p.(S300F)	HTTM (TMD4)	18.1 s [12–15 s]	1.2 [0.8–1.2]	PTT: 43.3 s [25–41 s]	64% [60%–150%]	108% [60%–160%]	62% [60%–160%]	33% [60%–160%]	no
9	p.(V255M)	HTTM	p.(S300F)	HTTM (TMD4)	21.6 s [12–15 s]	1.9 [0.8–1.2]	32.1 s [25–41 s]	43% [60%–150%]	31% [60%–160%]	56% [60%–160%]	18% [60%–160%]	no
10	p.(R83W)	HTTM	p.(Q374X)	TMD5	21–31 s [11–14 s]	-	-	20% [65%–150%]	74–117% [55%–185%]	48–71% [50%–180%]	20–22% [65%–185%]	no
11	p.(R83W)	HTTM	p.(Q374X)	TMD5	21–31 s [11–14 s]	-	-	18% [65%–150%]	88% [55%–185%]	56% [50%–180%]	18% [65%–185%]	no
12	p.(M174R)	HTTM	p.(I532T)	RmlC-like	-	2.76 [0.9–1.14]	ratio 1.44 [0.83–1.18]	20% [70%–131%]	34% [69%–134%]	42% [71%–139%]	20% [70%–13%]	no
13	p.(G72_L124del)	HTTM (TMD1 and -2)	p.(R485P)	near PP-BS	-	-	-	21% *	42% *	nd *	36%*	no
14	p.(W501S)	PP-BS	p.(W501S)	PP-BS	>100 s	-	PTT: >100 s	nd *	<1% *	9% *	26% *	yes
15	c.214+1G>T (splice)	HTTM (TMD1)	c.1609+3A>G (splice)	near PP-BS + RmlC-like	69.8 s [8.4–12.0 s]	7	45 s [21–33 s]	2% [70%–130%]	3% [65%–140%]	4% [65%–140%]	<3% [60%–130%]	yes
16	p.(W493S)	near PP-BS	p.(W493S)	near PP-BS	-	1.81 [0.8–1.2]	-	66% [90%–150%]	26% [90%–150%]	70% [90%–150%]	15 [90%–150%]	no
17	p.(R476C)	near PP-BS	-	-	-	1.97 [0.8–1.2]	-	38% [90%–150%]	50% [90%–150%]	103% [90%–150%]	20% [90%–150%]	no
18	p.(R476H)	near PP-BS	-	-	-	2.19 [0.8–1.2]	-	38% [90%–150%]	62% [90%–150%]	90% [90%–150%]	17% [90%–150%]	no
19	p.(Q374X)	TMD5	p.(G537A)	RmlC-like	-	1.7 [0.8–1.2]	-	20% [90%–150%]	74% [90%–150%]	48% [90%–150%]	20% [90%–150%]	no
20	p.(Q374X)	TMD5	p.(G537A)	RmlC-like	-	1.9 [0.8–1.2]	-	18% [90%–150%]	88% [90%–150%]	56% [90%–150%]	18% [90%–150%]	no
21	p.(H404P)	Glu-BS	p.(R485P)	near PP-BS	-	-	-	35% *	37% *	54% *	13%*	no
22	p.(W315X)	HTTM	p.(R485P)	near PP-BS	-	-	-	30% *	37% *	53% *	29% *	no
23	p.(R204C)	HTTM	p.(R204C)	HTTM	-	-	-	-	15% (after VK R/) *	-	6% *	yes
24	p.(R204C)	HTTM	p.(R204C)	HTTM	-	-	-	-	31% *	-	20% *	no
25	p.(R83P)	HTTM	p.(R83P)	HTTM	-	1.7 *	-	27% *	77% *	56% *	33% *	no
26	p.(R204C)	HTTM	p.(R204C)	HTTM	-	-	-	-	-	nd *	nd *	yes
27	p.(W157R)	HTTM (TMD3)	c.2085-5T>C (splice)	near Gla domain	-	-	-	-	54% *	-	28% *	no
28	p.(S284P)	HTTM	p.(W315X)	HTTM	-	-	-	41% *	27% *	nd *	18% *	no
29	p.(G125R)	HTTM (TMD2)	p.(D534V)	near PP-BS + RmlC-like	-	-	-	30% *	49% *	nd *	28% *	no
30	p.(W501S)	PP-BS	p.(W501S)	PP-BS				95% *	35% *	nd *	30% *	yes
31	p.(F74_G125del)	HTTM (TMD1 and -2)	p.(F74_G125del)	HTTM (TMD1 and -2)	-	-	-	-	-	-	-	no VKCFD1
32	p.(F74_G125del)	HTTM (TMD1 and -2)	p.(F74_G125del)	HTTM (TMD1 and -2)	100% [80%–100%]	-	PTT: 33.2 s [25–45 s]	76.2% [70%–80%]	86.5% [>60%]	70.5% [64%–84%]	67.1% [53%–122%]	no VKCFD1
33	p.(F74_G125del)	HTTM (TMD1 and -2)	p.(F74_G125del)	HTTM (TMD1 and -2)	100% [80%–100%]	-	PTT: 36.2 s [25–45 s]	73.2% [70%–80%]	94.8% [>60%]	68.7% [64%–84%]	72.4% [53%–122%]	no VKCFD1
34	p.(F74_G125del)	HTTM (TMD1 and -2)	p.(F74_G125del)	HTTM (TMD1 and -2)	100% [80%–100%]	-	PTT: 38.5 s [25–45 s]	78.8% [70%–80%]	119.3% [>60%]	69.8% [64%–84%]	58.3% [53%–122%]	no VKCFD1
35	p.(F74_G125del)	HTTM (TMD1 and -2)	p.(F74_G125del)	HTTM (TMD1 and -2)	100% [80%–100%]	-	PTT: 32.6 s [25–45 s]	74.1% [70%–80%]	126.7% [>60%]	69% [64%–84%]	66.8% [53%–122%]	no VKCFD1
36	p.(F74_G125del)	HTTM (TMD1 and -2)	p.(F74_G125del)	HTTM (TMD1 and -2)	94.7% [80%–100%]	-	PTT: 43.1 s [25–45 s]	73.1% [70%–80%]	76.3% [>60%]	70.4% [64%–84%]	71.2% [53%–122%]	no VKCFD1
37	p.(F74_G125del)	HTTM (TMD1 and -2)	p.(F74_G125del)	HTTM (TMD1 and -2)	100% [80%–100%]	-	PTT: 31.5 s [25–45 s]	83% [70%–80%]	79% [>60%]	76.9% [64%–84%]	58% [53%–122%]	no VKCFD1
38	p.(F74_G125del)	HTTM (TMD1 and -2)	p.(F74_G125del)	HTTM (TMD1 and -2)	-	-	-	-	-	-	-	no VKCFD1
39	p.(F74_G125del)	HTTM (TMD1 and -2)	p.(F74_G125del)	HTTM (TMD1 and -2)	100% [80%–100%]	-	PTT: 37.8 s [25–45 s]	77.8% [70%–80%]	73.7% [>60%]	-	55.9% [53%–122%]	no VKCFD1
40	p.(F74_G125del)	HTTM (TMD1 and -2)	p.(F74_G125del)	HTTM (TMD1 and -2)	-	-	-	-	-	-	-	no VKCFD1
41	p.(F74_G125del)	HTTM (TMD1 and -2)	p.(F74_G125del)	HTTM (TMD1 and -2)	96.4% [80%–100%]	-	PTT: 38.9 s [25–45 s]	78.1% [70%–80%]	81.9% [>60%]	70% [64%–84%]	67.7% [53%–122%]	no VKCFD1
42	p.(F74_G125del)	HTTM (TMD1 and -2)	p.(F74_G125del)	HTTM (TMD1 and -2)	100% [80%–100%]	-	PTT: 38.9 s [25–45 s]	74.2% [70%–80%]	129.3% [>60%]	68.5% [64%–84%]	72.8% [53%–122%]	no VKCFD1
43	p.(F74_G125del)	HTTM (TMD1 and -2)	p.(F74_G125del)	HTTM (TMD1 and -2)	100% [80%–100%]	-	PTT: 31.8 s [25–45 s]	76% [70%–80%]	106% [>60%]	68.6% [64%–84%]	71.5% [53–122%]	no VKCFD1
44	/(14 bp del I1)	-	/(14 bp del I1)	-	>100 s	-	>150 s	0.35 U/mL *	0.08 U/mL *	nd ^†^	0.21 U/mL *	yes
45	/(14 bp del I1)	-	/(14 bp del I1)	-	30 s	-	38 s	0.09 U/mL *	0.21 U/mL *	0.59 U/mL	0.17 U/mL *	no
46	p.(W493C)	near PP-BS	p.(R704X)	near Gla-domain	>100 s [12.8 s]	-	-	3% *	2% *	-	3% *	yes
47	p.(D15_F71del)	N-terminus + HTTM (TMD1)	p.(D15_F71del)	N-terminus + HTTM (TMD1)	98.9 s	9	53.1 s	2% *	1.7% *	4.7% *	2% *	no

14 bp del I1: 14 base pair deletion intron 1; aPTT: activated partial thromboplastin time; FII: coagulation factor II; FVII: coagulation factor VII; FIX: coagulation factor IX; FX: coagulation factor X; Glu-BS: glutamate binding site; HTTM: horizontally transferred transmembrane domain; Id: identification number; INR: international normalized ratio; nd: not described; PT: prothrombin time; PP-BS: propeptide binding site; RmlC: deoxythymidine-6-deoxy-d-xylo-4-hexulose 3,5 epimerase (EC5.1.3.13); RmlC-like: RmlC-like jelly roll fold; splice: splice site mutation; TMD: transmembrane domain; VK R/: vitamin K therapy; * no reference values in original article or no values, but clearly stated in full-text as deficient.^†^ deficient factor IX confirmed at 9 years of age.

**Table 9:** Vitamin K treatment in VKCFD1 patients with *GGCX* mutations. This table gives an overview of all patients who received vitamin K treatment for their coagulation factor deficiency and the response to treatment. For each patient, an overview of the PT, aPTT, coagulation factor function (percent of normal activity or in U/dL or U/mL) prior to therapy is given (in italics) and extrahepatic (non-hemostatic) before treatment parameters are shown (in italics), if determined. Further, the clinical response to treatment is mentioned. For P21, P27, P28, and P29, the response to treatment for the ucOC/cOC ratio is given, but the specific vitamin K dose at the time of blood sampling is not mentioned in the original article. Reference values are stated between brackets if they were mentioned in the original article.

Id	VK R/(age)	PT	INR	aPTT	FII	FVII	FIX	FX	PC	ProS	Extrahepatic Proteins	New Bleeding Episodes
**1**	*before treatment*	*>120 s*	*-*	*>180 s*	*2 U/dL [77–125 U/dL]*	*3 U/dL [63–139 U/dL]*	*8 U/dL [63–155 U/dL]*	*2 U/dL [55–160 U/dL]*	*-*	*-*	*-*	
	10 mg sc/week	-	-	-	18 U/dL [77–125 U/dL]	25 U/dL [63–139 U/dL]	37 U/dL [63–155 U/dL]	15 U/dL [55–160 U/dL]	45 U/dL [65–146 U/dL]	34 U/dL [74–126 U/dL]	-	yes
**2**	*before treatment*	*-*	*-*	*-*	*24 U/dL [77–125 U/dL]*	*23 U/dL [63–139 U/dL]*	*8 U/dL [63–155 U/dL]*	*20 U/dL [55–160 U/dL]*	*42 U/dL [65–146 U/dL]*	*35 U/dL [74–126 U/dL]*		
	10 mg sc/week	-	2–3.5	-	45 U/dL [77–125 U/dL]	43 U/dL [63–139 U/dL]	89 U/dL [63–155 U/dL]	27 U/dL [55–160 U/dL]	73 U/dL [65–146 U/dL]	35 U/dL [74–126 U/dL]	-	no
**3**	*before treatment*	nd *	nd *	nd *	nd *	nd *	nd *	nd *	nd *	nd *		
	10 mg sc/week	-	-	-	31 U/dL [77–-125 U/dL]	23 U/dL [63–139 U/dL]	55 U/dL [63–155 U/dL]	17 U/dL [55–160 U/dL]	84 U/dL [65–146 U/dL]	28 U/dL [74–126 U/dL]	-	no
**4**	*before treatment*	nd *	nd *	nd *	nd *	nd *	nd *	nd *	nd *	nd *		
	10 mg sc/week	-	-	-	24 U/dL [77–125 U/dL]	47 U/dL [63–139 U/dL]	33 U/dL [63–155 U/dL]	16 U/dL [55–160 U/dL]	71 U/dL [65–146 U/dL]	57 U/dL [74–126 U/dL]	-	no
**5**	*before treatment*	*49.5 s [11.5 s]*	*-*	*60 s [30 s]*	9% *	6% *	7% *	5% *	*-*	*-*		
	10 mg/d IM 2 weeks (VK1)	no effect	-	no effect	-	-	-	-	-	-	-	?
**7**	*before treatment*	*11% [70–100%]*	*-*	*ratio 1.65 [0.84–1.21]*	*26% [50%–150%]*	*<1% [50%–150%]*	*12% [50%–150%]*	*13% [50%–150%]*	*7% [70%–140%]*	*10% [60%–120%]*	*dp-ucMGP: 2387 pM [35–546 pM]*	
	a. 10 mg/day po 3 months (VK1)	36% [70–100%]	-	ratio: 1.24 [0.84–1.21]	46% [50%–150%]	21% [50%–150%]	53% [50%–150%]	27% [50%–150%]	13% [70%–140%]	6% [60%–120%]	dp-ucMGP 2750 pM [35–546 pM]	no
	b. 20 mg/d po > 1 y ^†^ (VK1)	42% [70–100%]	-	ratio 1.02 [0.84–1.21]	38% [50%–150%]	21% [50%–150%]	54% [50%–150%]	31% [50%–150%]	21% [70%–140%]	8% [60%–120%]	dp-ucMGP 2407 pM [35–546 pM]	no
**10**	*before treatment*	*21–31 s [11–14 s]*	*-*	*-*	*20% [65%–150%]*	*74*–*117%* *[55%–185%]*	*48*–*71%* *[50%–180%]*	*20*–*22%* *[65%–185%]*	*-*	*-*	*-*	
	a. 1× parenteral (VK1)	-	normal	-	-	-	-	-	-	-	-	no
	b. 10 mg po/d 2 weeks (VK1)	-	-	-	normal	-	normal	normal	-	-	-	no
	c. 5 mg/d (VK1)	14.9 s [12.3–14.6 s]	-	PTT: 25.9 s [27.3–35.3]	-	-	-	-	-	-	-	no
**12**	*before treatment*	*-*	*2.76 [0.9–1.14]*	*ratio 1.44 [0.83–1.18]*	*20% [70%–131%]*	*34% [69%–134%]*	*42% [71%–139%]*	*20% [70%–135%]*	*47% [65%–132%]*	*-*	*-*	
	po	-	1.94 [0.9–1.14]	ratio: 1.26 [0.83–1.18]	24% [70%–131%]	34% [69%–134%]	54% [71%–139%]	18% [70%–135%]	47% [65%–132%]	-	-	no
	iv	-	1.98 [0.9–1.14]	ratio: 1.16 [0.83–1.18]	20% [70%–131%]	35% [69%–134%]	54% [71%–139%]	18% [70%–135%]	47% [65%–132%]	67% [62%–131%]	-	no
**13**	*before treatment*	*-*	*-*	*-*	21% *	42% *	nd *	36% *	*-*	*-*	*-*	*Incidental finding*
	2 mg/d 6 weeks	-	-	-	40%	62%	-	65%	-	-	ucOC: >9.4 μg/L *	no
**14**	*before treatment*	*>100 s*	*-*	*PTT: >100 s*	nd *	<1% *	9% *	26% *	*-*	*-*	*-*	
	5 mg/d po (VK1)	-	-	-	-	-	-	-	-	-	-	?
**15**	*before treatment*	*69.8 s [8.4–12.0s]*	*7*	*45 s [21–33 s]*	*2% [70%–130%]*	*3% [65%–140%]*	*4% [65%–140%]*	*<3% [60%–130%]*	*-*	*-*	*-*	
	a. 5 mg/2 days po	16.1 s [8.4–12.0s]	-	29 s [21–33 s]	20% [70%–130%]	22% [65%–140%]	46% [65%–140%]	23% [60%–130%]	45% [70%–130%]	9% [65%–130%]	-	no
	b. 30 mg/d po	-	-	-	32% [70%–130%]	43% [65%–140%]	58% [65%–140%]	25% [60%–130%]	-	-	-	no
**21**	*before treatment*	*-*	*-*	*-*	35% *	37% *	54% *	13% *	*56%*	*37%*	*ratio ucOC/cOC: 30.3 [1.2]*	
	a. 70 mg (38 y)	-	-	-	71%	69%	90%	28%	63%	48%	ratio ucOC/cOC: 11.9 [1.2]	?
	b. 70 μg (47 y)	-	-	-	77%	79%	84%	30%	81%	-	-	?
	c. 105 μg (47 y)	-	-	-	94%	76%	100%	37%	-	-	-	?
**22**	*before treatment*	*-*	*-*	*-*	30% *	37% *	53% *	29% *	*-*	*-*	*-*	*incidental finding*
	a. 6 mg (14 y)	-	-	-	34%	53%	63%	39%	-	-	-	no
	b. 14 mg (20 y)	-	-	-	41%	51%	59%	26%	30%	17%	-	no
	c. 70 mg (20 y)	-	-	-	53%	59%	91%	39%	-	-	-	no
**23**	*before treatment*	*-*	*-*	*-*	*-*	15% (VK R/) *	*nd*	6% *	*-*	*-*	*-*	
	a. 1 × 1 mg (10 days)	-	-	-	-	-	-	30%	-	-	-	?
	b. 1 × 1 mg (17 days)	-	-	-	-	-	-	22%	-	-	-	?
	c. 30 mg (7 y)	-	-	-	-	80%	-	42%	-	-	-	?
	d. 50 mg (11 y)	-	-	-	34%	47%	40%	33%	55%	27%	-	?
	e. 90 mg (11 y)	-	-	-	51%	68%	54%	51%	69%	35%	-	?
**24**	*before treatment*	*-*	*-*	*-*	*-*	31% *	*-*	20% *	*-*	*-*	*-*	
	a. 30 mg (10 y)	-	-	-	-	65%	-	41%	-	-	-	?
	b. 50 mg (14 y)	-	-	-	38%	38%	46%	40%	47%	25%	-	?
	c. 90 mg (14 y)	-	-	-	69%	80%	67%	71%	67%	38%	-	?
**25**	*before treatment*	*-*	1.7 *	*-*	27% *	77% *	56% *	33% *	*-*	*-*	*-*	
	a. 1 × 3 mg (1 y)	-	1.4	-	46%	60%	61%	50%	-	-	-	?
	b. 17.5 mg (3 y)	-	1.3	-	61%	65%	51%	67%	-	-	-	?
**26**	*before treatment*	*-*	*-*	*-*	*-*	*-*	nd *	nd *	*-*	*-*	*-*	
	a. 140 mg (3 months)	-	-	174 s	-	-	-	-	-	-	-	?
	b. 21 mg (1 y)	-	1.6	-	-	-	37%	-	-	-	-	?
	c. 23.3 mg (3 y)	-	2.4	-	35%	40%	49%	25%	27%	10%	-	?
	d. 23.3 mg (4 y)	-	1.7	-	-	-	-	-	-	-	-	?
**27**	*before treatment*	*-*	*-*	*-*	*-*	54% *	*-*	28% *	*-*	*-*	*ratio ucOC/cOC: 66.9 [1.2]*	
	a. 140 mg (13 y)	-	1.2	-	89%	108%	121%	55%	53%	36%	ratio ucOC/cOC: 39.3 [1.2]	?
	b. 140 mg (14 y)	-	1.1	-	87%	83%	131%	53%	52%	59%	-	?
**28**	*before treatment*	*-*	*-*	*-*	41% *	27% *	nd *	18% *	*76%*	*-*	*ratio ucOC/cOC: 4.3 [1.2]*	
	a. 21 mg (12 y)	-	-	-	62%	60%	-	48%	106%	-	ratio ucOC/cOC: 3.9 [1.2]	?
	b. 21 mg (13 y)	-	-	-	60%	67%	98%	31%	-	-	-	?
	c. 70 mg (13 y)	-	-	-	76%	65%	-	57%	99%	-	-	?
**29**	*before treatment*	*-*	*-*	*-*	30% *	49% *	nd *	28% *	*-*	*-*	*ratio ucOC/cOC: 25.8 [1.2]*	
	70 mg (5 y)	-	-	-	56%	75%	63%	41%	30%	16%	ratio ucOC/cOC: 9.9 [1.2]	?
**30**	*before treatment*	21%	3.8 *	41 s [28 s]	9% *	35% *	nd *	30% *	-	-	-	
	a. 2 × 5 mg/d 3 days	40%	2	36 s [28 s]	13%	69%	13%	38%	38%	-	-	no
	b. multiple doses ^‡^	no abnormalities at 3.5 y (2 years after cessation treatment)									-	no
**44**	*before treatment*	*>100 s*	*-*	*>150 s*	*0.11 U/mL* ^§^	*0.12 U/m* ^§^	*0.30 U/mL* ^§^	*0.09 U/mL* ^§^	*-*	*-*	*-*	
	2 mg po	-	-	-	good response	modest response	good response	good response	-	-	-	?
**45**	*before treatment*	*30 s*	*-*	*38 s*	0.09 U/mL *	0.21 U/mL *	*0.59 U/mL*	0.17 U/mL *	*-*	*-*	*-*	*incidental finding*
	1 mg iv	-	-	-	good response	good response	no response	no response	-	-	-	?
**46**	*before treatment*	*>100 s [12.8]*	*-*	*-*	3% *	2% *	*-*	3% *	*-*	*-*	*-*	
	infusion	14.8 s [12.8 s]	-	-	72%	62%		62%	-	-	-	no
**47**	*before treatment*	*98.9 s*	9 *	*53.1s*	2% *	1.7% *	4.7% *	2% *	*-*	*-*	ratio ucOC/cOC: 84.5 *	
	VK1: 5–10 mg/d po or iv (VK1)	95.4 s ± 11.9 s	8.9 ± 0.8	40.0 s ± 7.3 s	3.7% ± 0.9%	3.2% ± 1.7%	9.1% ± 2.8%	4.7% ± 2.1%	4.1% ± 2.1%	5.6% ± 0.8%	ratio ucOC/cOC: 9.5 *	yes

?: not mentioned (in column “new bleeding episodes”); 14 bp del I1: 14 base pair deletion intron 1; (a) PTT: (activated) partial thromboplastin time; d: day (only in column “VK R/”), cOC: gamma-carboxylated osteocalcin; dp-ucMGP: desphospho-uncarboxylated matrix gla protein; FII: coagulation factor II; FVII: coagulation factor VII; FIX: coagulation factor IX; FX: coagulation factor X; Id: identification number; IM: intramuscular; INR: international normalized ratio; nd: not described; VK R/: vitamin K therapy; PC: protein C; po: per os (oral); ProS: protein S; PT: prothrombin time; s: seconds (only in columns “PT and aPTT”); sc: subcutaneous; TMD: transmembrane domain; VK: vitamin K; ucOC: uncarboxylated osteocalcin; y: year (only in column “VK R/”); ^†^ therapy also included: +41 mg MK-4/day and 2 mg MK-7/day for 6 months; MK-4 and MK-7 are homologue of vitamin K2; * no reference values in original article or no values, but clearly stated in full-text as deficient; ^‡^ 2 × 5 mg VK IM/week (2 weeks), then 5 mg VK IM/week (4 weeks), then 5 mg VK IM/month (16 months), then cessation therapy with continued normal coagulation parameters; ^§^ deficient factor IX confirmed at 9 years of age.

## Supplementary Materials

Supplementary materials can be found at www.mdpi.com/1422-0067/18/2/240/s1.

## Abbreviations

AA	amino acid
ABCC6	ATP-binding cassette, subfamily C, member 6
ADCL1	autosomal dominant cutis laxa, type 1
aPTT	activated partial thromboplastin time
ARSE	arylsulphatase E
ATP	adenosine triphosphate
AXL	AXL receptor tyrosine kinase
cOC	gamma-carboxylated osteocalcin
EC	Escherichia coli
EF	elastic fiber
ER	endoplasmic reticulum
FII	coagulation factor II
FIX	coagulation factor IX
FVII	coagulation factor VII
FX	coagulation factor X
Gas6	growth arrest-specific 6
GGCX	gamma-glutamyl carboxylase
Gla	gamma-carboxyglutamate
Glu	glutamate
GRP	gla-rich protein
HGVS	Human Genome Variation Society
HTTM	horizontally transferred transmembrane domain
INR	international normalized ratio
kDa	kiloDalton
MerTK	Mer tyrosine kinase proto-oncogene
MGP	matrix gla protein
OC	osteocalcin
OMIM	online mendelian inheritance in man
P[1–45]	patient identification
PRGP	proline-rich gla protein
PRISMA-P	preferred reporting items for systematic reviews and meta-analysis protocols
ProS	protein S
PT	prothrombin time
PXE	pseudoxanthoma elasticum
RmlC	deoxythymidine-6-deoxy-d-xylo-4-hexulose 3,5 epimerase; EC5.1.3.13
RmlC-like	RmlC-like jelly roll fold
RTK	receptor tyrosine kinase
SNP	single nucleotide polymorphism
TMD	transmembrane domain
TMG	transmembrane gla protein
ucOC	uncarboxylated osteocalcin
VK	vitamin K
VKDP	vitamin K-dependent protein
VKORC1	vitamin K epoxide epoxide reductase complex, subunit 1
WBSCR	Williams-Beuren syndrome critical region
