# Supplementary material for: GGCX-Associated Phenotypes: An Overview in Search of Genotype-Phenotype Correlations
Source: Int J Mol Sci. 2017 Jan 25;18(2):240. doi: 10.3390/ijms18020240 (PMC5343777; doi:10.3390/ijms18020240)
Supplement: Supplementary file 1 [file ijms-18-00240-s001.zip › ijms-161614-Table S3.pdf]

Supplementary Materials: GGCX-Associated Phenotypes: An Overview in Search of Genotype-Phenotype Correlations

Eva Y. G. De Vilder, Jens Debacker and Olivier M. Vanakker

Table S3. Multiple sequence alignment of the GGCX amino acid residues 459-508 (NM000812.2) in 11 different species.

Transcript Number (Species Number)	Number of First Amino Acid	GGCX Sequence	Number of Last Amino Acid
NP_000812.2 (1)	459	NVTEPQIYFDIWVSINDRFQQRIFDPRVDIVQAAWSPFQRTSWVQPLLMD	508
XP_515586.2 (2)	459	NVTEPQIYFDIWVSINDRFQQRIFDPRVDIVQAAWSPFQRTSWVQPLLMD	508
XP_001086474.1 (3)	459	NVTEPQIYFDIWVSINDRFQQRIFDPRVDIVQAAWSPFQRTSWVQPLLMD	508
XP_532979.3 (4)	459	NVTEPQIYFDIWVSINDRFQQRIFDPRVDIVQAAWSPFQRTPWLQPLLMD	508
NP_776491.1 (5)	459	NVTEPQIYFDIWVSINDRFQQRIFDPRVDIVQAAWSPFQRTPWLQPLLMD	508
NP_062776.1 (6)	459	NVTEPQIYFDIWVSINDRFQQRLFDPRVDIVQAVWSPFQRTPWVQPLLMD	508
NP_113944.1 (7)	459	NVTEPQIYFDIWVSINDRFQQRLFDPRVDIVQAVWSPFRRTPWVQPLLMD	508
XP_003199342.1 (8)	179	NISDPEIYFDIWVSINDRFQQRIFDPHVDIVKADWSPFRPNPWLMPLLVD	228
NP_001163323.1 (9)	462	GR-NISIYFDIWCSMNGRFQQRSFDPREDLLRAKWSPFESTSWSLPLLNE	510

The arginine-residue at position 476 (yellow highlight) is part of a highly conserved 7 AA-long sequence (N-NDRFQQR-C) and the tryptophan-residue at position 493 (green highlight) is highly conserved among different species (11/11); 1: *homo sapiens*—human; 2: *pan troglodytes*—common chimpanzee; 3: *macaca mulatta*—*rhesus macaque*; 4: *canis lupus familiaris*—dog; 5: *bos taurus*—European cattle; 6: *mus musculus*—house mouse; 7: *rattus norvegicus*—brown rat; 8: *danio rerio*—zebrafish; 9: *drosophilia melanogaster*—fly; 10: *anopheles gambiae str. PEST*—mosquito, 11: *xenopus tropicalis*—western clawed frog.
